# The effect of subgroup homogeneity of efficacy on contribution in public good dilemmas

**DOI:** 10.1371/journal.pone.0201473

**Published:** 2018-07-31

**Authors:** Paton Pak Chun Yam, Gary Ting Tat Ng, Wing Tung Au, Lin Tao, Su Lu, Hildie Leung, Jane M. Y. Fung

**Affiliations:** 1 Department of Psychological and Behavioural Science, London School of Economics and Political Science, London, England; 2 Department of Psychology, The Chinese University of Hong Kong, Hong Kong SAR, China; 3 Department of Sociology, Peking University, Beijing, China; 4 Department of Human Resource and Organizational Behavior, University of International Business and Economics, Beijing, China; 5 Department of Applied Social Sciences, The Hong Kong Polytechnic University, Hong Kong SAR, China; Middlesex University, UNITED KINGDOM

## Abstract

This paper examines how to maximize contribution in public good dilemmas by arranging people into homogeneous or heterogeneous subgroups. Past studies on the effect of homogeneity of efficacy have exclusively manipulated group composition in their experimental designs, which might have imposed a limit on ecological validity because group membership may not be easily changed in reality. In this study, we maintained the same group composition but varied the subgroup composition. We developed a public good dilemmas paradigm in which participants were assigned to one of the four conditions (high- vs. low-efficacy; homogeneous vs. heterogeneous subgroup) to produce their endowments and then to decide how much to contribute. We found that individuals in homogeneous and heterogeneous subgroups produced a similar amount and proportion of contribution, which was due to the two mediating effects that counteracted each other, namely (a) perceived efficacy relative to subgroup and (b) expectation of contribution of other subgroup members. This paper demonstrates both the pros and cons of arranging people into homogeneous and heterogeneous subgroups of efficacy.

## Introduction

Cooperation is vital to the welfare of organizations, communities, and states in the modern society [1, 2] in promoting team work, conservation of natural resources, mitigation of green-house-gas emissions, etc. A vast amount of theoretical and empirical work has been devoted to understanding cooperation through various outlooks ranging from individual difference, cultural, to evolutionary perspectives [2–10]. Although most of the traditional economic models assume that all human beings are rational, self-interested agents, and do not prioritize social goals, both laboratory experiments and real-life examples have indicated that people cooperate to a non-negligible extent, e.g., [11, 12]. Many of those works study cooperation in the context of public good dilemma (PGD), a situation where the whole group can benefit the most if all its members contribute everything to the common pool, but the self-maximizing strategy is to free ride and not to contribute anything [13, 14].

One important factor that was found to exert an influence on cooperation in PGD is the group composition [15, 16]. One way to characterize the group composition is group homogeneity and heterogeneity (or group diversity), which are defined as the degree of similarity and difference of attributes of group members respectively [17, 18]. Research on group homogeneity and heterogeneity can largely be classified into a social categorization perspective that examines how ingroup/outgroup differences may disrupt the group processes; and an information perspective that stipulates how diversity in knowledge and expertise may facilitate group performance [19]. Past studies on organizational effectiveness have investigated the effects of group homogeneity and heterogeneity based on attributes ranging from surface level diversity (e.g., demographic characteristics of race, age, and gender) to deep level diversity (e.g., attitude, personality, and values) [20], and such group homogeneity and heterogeneity can have both positive and negative effects on organizational outcomes [21, 22]. This paper focuses on the effect of subgroup homogenous and heterogenous distributions of efficacy on contribution in social dilemmas.

Suppose we have a 12-person work team with six high-efficacy and six low-efficacy members respectively, and they have to work on a specific task in a subgroup of three. How can we split the team in order to maximize their contributions? On the one hand, we can split the team into four homogeneous subgroups, with two subgroups each having three high-efficacy members; and the other two subgroups each having three low-efficacy members. On the other hand, we can also split the team into four heterogeneous subgroups, with two subgroups each having one high-efficacy member and two low-efficacy members; and the other two subgroups each having two high-efficacy members and one low-efficacy member. While previous experimental studies have shown the effect of homogeneous distribution of efficacy on contribution in PGD, e.g., [23, 24]; their findings may not be sufficient to address the above question. This is because the way they manipulated the group composition of efficacy changed the total number of high- and low-efficacy individuals (i.e., the “total” efficacy) in a group. In our case, just like most situations in daily life, the “total” efficacy of a group remains the same; we are stuck with the people that we have. The only way to change group homogeneity is to divide group members into subgroups. Therefore, the study reported in this paper aimed to examine whether and how we can maximize group contribution by strategically changing the subgroup composition in organizations, while keeping the total efficacy of group members unchanged.

In the following, we will first introduce the concept of “efficacy” that will be referred to throughout this paper. We will then summarize some past research on the relation between homogeneity of efficacy and contribution. After that, we will highlight the distinctive features of the present study, and finally we will propose two potential mediators–perceived efficacy and expectation of contribution–that could help explain the underlying mechanisms behind the aforementioned relations.

### Efficacy

Efficacy, criticality, and indispensability are similar constructs that have been used to describe an individual’s impact on PG provision in a PGD, e.g., [25–29]. Particularly, Yu et al. [29] proposed that Efficacy = Endowment × Efficiency, in which endowment is the resource that a person can contribute while efficiency is the impact brought about by each unit of endowment. For example, in a group project, a person is considered to have a high efficacy if he or she has much time available for the group project (endowment) and can accomplish much work—per hour spent (efficiency). Individuals’ efficacy varies with group size [30]. Keeping individuals’ endowment constant, as group size increases, the relative impact of each unit of endowment (efficiency) decreases, so as efficacy. In our study, we kept the “total” efficacy of the group unchanged, manipulating only the efficacy composition of each subgroup.

### Homogeneity of efficacy and group contribution

Past studies that have examined the effect of group homogeneity of efficacy on contribution focused on endowment heterogeneity (also termed as endowment asymmetry or resource inequality, e.g., [31, 32]). These studies yielded mixed findings. On the one hand, Aquino et al. [31] found that high resource inequality led to less cooperation in social dilemmas. They argued that, under high resource inequality, people with fewer resources tended to free ride because they perceived their contributions to be dispensable; while people with more resources also tended to free ride because they expected others to do so as well. Rapoport and Suleiman [33] also concluded from a step-level paradigm with provision threshold that heterogeneous groups were less successful in providing PG than homogeneous groups. Similarly, Cherry et al. [34] showed that contribution levels were significantly lower when groups had heterogeneous rather than homogeneous endowments, irrespective of whether the endowments were determined by the performance of a task or were just randomly assigned. Fung and Au [24] also found that both the symmetrically heterogeneous and the hegemonic heterogeneous groups contributed less than homogeneous groups. In terms of proportion of contributions, Hargreaves Heap et al. [35] showed that individuals with high endowment contributed proportionally less in heterogeneous groups than in homogeneous groups.

On the other hand, there has also been research showing that heterogeneous groups contribute more [23]. For example, heterogeneity of endowment was shown to have a positive effect on aggregate contribution, but the effect was moderated by (a) whether communication was allowed; (b) whether participants received complete information about the payoff; and (c) whether the marginal return was the same for each member [36]. Another study showed that when a best-shot PGD was determined by the highest contribution rather than sum of all contributions, endowment heterogeneity resulted in better coordination as it provided a shared expectation among group members [37].

Still, Warr [38] illustrated mathematically that the distribution of resources should have no effect on the contribution of PG. In line with this prediction, Levati et al. [39] found that homogeneous groups had similar contribution compared to heterogeneous groups. Table 1 shows the summary of these previous findings.

**Table 1:** Summary of studies of the effect of homogeneity of efficacy on contribution in public good dilemmas.

Authors	Year	Sample size	Group size	Provision threshold	Fixed/Continuous return	Major findings
Aquino et al.	1992	96	4	Yes	Continuous	Resource inequality led to decreased contribution.
Chan et al.	1996	75	3	No	Continuous	Endowment heterogeneity increased contribution for high, but not low degree of heterogeneity.
Chan et al.	1999	72	3	No	Continuous	Endowment heterogeneity increased aggregate contribution, but the effect was moderated by whether communication was allowed, whether participants received complete information about the payoff, and whether the marginal return was the same for each member.
Cherry et al.	2005	124	4	No	Continuous	Contribution levels were significantly lower when groups had heterogeneous rather than homogeneous endowments, irrespective of whether the endowments were earned or randomly assigned.
Cherry et al.	2013	192	4	No	Continuous	In a best-shot PG in which the provision level is determined by the highest contribution instead of the sum of all contributions, endowment heterogeneity resulted in better coordination.
Fung & Au (Study 1)	2014	96	3	No	Continuous	Both the symmetrically heterogeneous and the hegemonic heterogeneous groups contributed less than homogeneous groups.
Hargreaves Heap et al.	2016	210	3	No	Continuous	Individuals with high endowment contributed proportionally less in heterogeneous groups than in homogeneous groups.
Levati et al.	2007	328	4	No	Continuous	No significant differences in contribution between homogeneous and heterogeneous groups.
Rapoport & Suleiman	1993	60	5	Yes (vary across conditions)	Fixed	Heterogeneous groups were less successful in providing public goods than homogeneous groups were.

### The present study

In the present study, participants role-played a member in a work team of a social enterprise that comprised of six high-efficacy and six low-efficacy members. With the explicit knowledge of this group composition, each participant was either assigned into a three-person homogeneous subgroup (i.e., either all high-efficacy or all low-efficacy members) or a three-person heterogeneous subgroup (i.e., either two high- and one low-efficacy members or two low- and one high-efficacy members). Participants were required to produce cell phone straps and decide how much to contribute to the social enterprise.

There are two main objectives of the present study. The first is to examine whether and how we can maximize contribution by strategically changing the subgroup compositions into either homogeneous subgroups or heterogeneous subgroups while keeping the composition of group members unchanged. With this goal in mind, our study is designed with two features that distinguish itself from previous research. Firstly, we manipulated the homogeneity of efficacy by varying the subgroup configurations. Although previous research also compared the effects of homogeneity and heterogeneity of efficacy on contribution, they focused on the effects with respective to *groups* rather than s*ubgroups* e.g., [23, 31, 34]. Their studies thus manipulated the homogeneity of efficacy by changing the initial endowment of group members. The second and a more important feature lies in how the interdependence of task outcomes are implicated for subgroups as well as groups [40]. Past research compared the contributions of homogeneous groups and heterogeneous groups by having the PG shared among people within their own homogeneous or heterogeneous group *only*. In other words, a participant’s outcome is independent of the contributions of other groups. In the current study, however, all subgroups were nested within the same group. Participants, though stayed in the subgroup, received the same PG that was jointly determined by all members in the group, including members of other subgroups. Therefore, participants’ outcome was contingent on the contributions of their own, as well as other subgroups.

The second objective of the present study is to understand the underlying mechanisms that may explain why subgroup homogeneity can increase or decrease contribution. Specifically, we test two potential mediators that are known to influence cooperation in social dilemmas, namely perceived efficacy of *oneself* and expectation of contribution towards *others*.

### Underlying mechanisms

#### Perceived efficacy

We distinguish between *absolute efficacy* and *perceived efficacy*. We define absolute efficacy as the actual impact on the PG provision and perceived efficacy as the personal belief about one’s impact relative to the affiliated group or subgroup. Specifically, in a symmetric PGD with homogeneous endowment, the absolute efficacy of each individual increases when endowment increases, but the perceived efficacy relative to group members may not change because no one is comparatively more efficacious than the others. In the present study, we randomly assigned participants into either high or low absolute efficacy conditions. For simplicity, we use “high-efficacy individuals” and “low-efficacy individuals” to refer to the participants being assigned to high and low absolute efficacy conditions, respectively.

Perceived efficacy has been shown to be effective in inducing cooperation through enhancing a sense of social responsibility [41]. Research under the rubrics of efficacy and criticality all point to the same conclusion that a person who perceives a larger impact on PG provision contributes more. Beyond laboratory settings, perceived efficacy also influences various kinds of organizational behaviors, including job performance [42].

Various factors can influence cooperation rate through efficacy. For example, a large group size decreases cooperation drastically because it diminishes one’s perceived efficacy [43]. Kerr [30] also demonstrated that people in a small group reported higher perceived efficacy and hence cooperated more than those in a large group, despite the fact that their impacts on the outcome were made objectively the same in both the small and large groups.

While subgroup configuration does not affect the absolute efficacy of individuals, it does affect the perceived efficacy. Indeed, an individual’s perceived efficacy *relative to the group* may be different from that *relative to the subgroup*. Intuitively, subgroup configuration may not affect the perceived efficacy relative to the group because individuals should be aware of the fact that the efficacy levels of other members in the group remain the same, regardless of the subgroup composition. However, in terms of perceived efficacy relative to the *subgroup*, subgroup homogeneity matters. Among high-efficacy individuals, those in heterogeneous subgroups shall perceive higher efficacy relative to the subgroup than those in homogeneous subgroups, because they can make downward comparison with low-efficacy individuals within the same subgroup. On the contrary, among low-efficacy individuals, those in homogeneous subgroups shall perceive higher efficacy than those in heterogeneous subgroups because in homogeneous subgroups everyone is of low-efficacy and there is no upward comparison. We thus predict an absolute efficacy × subgroup homogeneity interaction effect on perceived efficacy, and higher perceived efficacy relative to the subgroup will in turn lead to higher contribution. Specifically, we hypothesize that high efficacy individuals will perceive higher efficacy and will hence contribute more in heterogeneous subgroups than in homogenous subgroups, whereas low efficacy individuals will perceive higher efficacy and hence contribute more in homogeneous subgroups than in heterogenous subgroups *(Hypothesis 1)*.

#### Expectation of contribution

Expectation of contribution refers to the amount that one expects the other members to contribute. Expectation of others’ contributions affect our own contribution. For instance, worrying that others are not going to contribute may induce fear of being a sucker. One may also develop a sense of greed to free ride on others if we expect others to contribute considerably [44, 45]. Furthermore, Pruitt and Kimmel’s Goal/Expectation Hypothesis [46] states that, in addition to adopting a mutual goal of cooperation, developing mutual expectation of cooperation is essential in enhancing cooperation. Indeed, previous research demonstrated that cooperation increased when others were expected to cooperate [47, 48].

A person with higher efficacy can potentially make a stronger impact on PG provision. Therefore, it is reasonable to expect that a person with higher efficacy will contribute more. For expectation of *group* contribution, subgroup homogeneity should not affect the expectation of contribution towards the group because the group has a fixed configuration (e.g., six high-efficacy and six low-efficacy members, as in the previous example). For expectation of *subgroup* contribution, however, subgroup homogeneity matters. Specifically, high-efficacy individuals in the homogeneous subgroups shall develop a higher expectation of subgroup contribution than their counterparts in the heterogeneous subgroups. This is because all members in high-efficacy homogeneous subgroups are of high-efficacy. On the contrary, low-efficacy individuals in heterogeneous subgroups shall develop a higher expectation of subgroup contribution than those in the homogeneous subgroups. This is because there is at least one other high-efficacy member who is supposed to contribute a lot. We thus predict an absolute efficacy × subgroup homogeneity interaction effect on expectation of subgroup contribution and a positive relation between higher expectation of subgroup and contribution. Specifically, high efficacy individuals will expect other subgroup members to contribute more and in turn they themselves will also contribute more in homogenous subgroups than in heterogenous subgroups, whereas low efficacy individuals in heterogenous subgroups will expect other subgroup members to contribute more and in turn they themselves will also contribute more than their homogenous counterparts *(Hypothesis 2)*.

**Fig 1:**
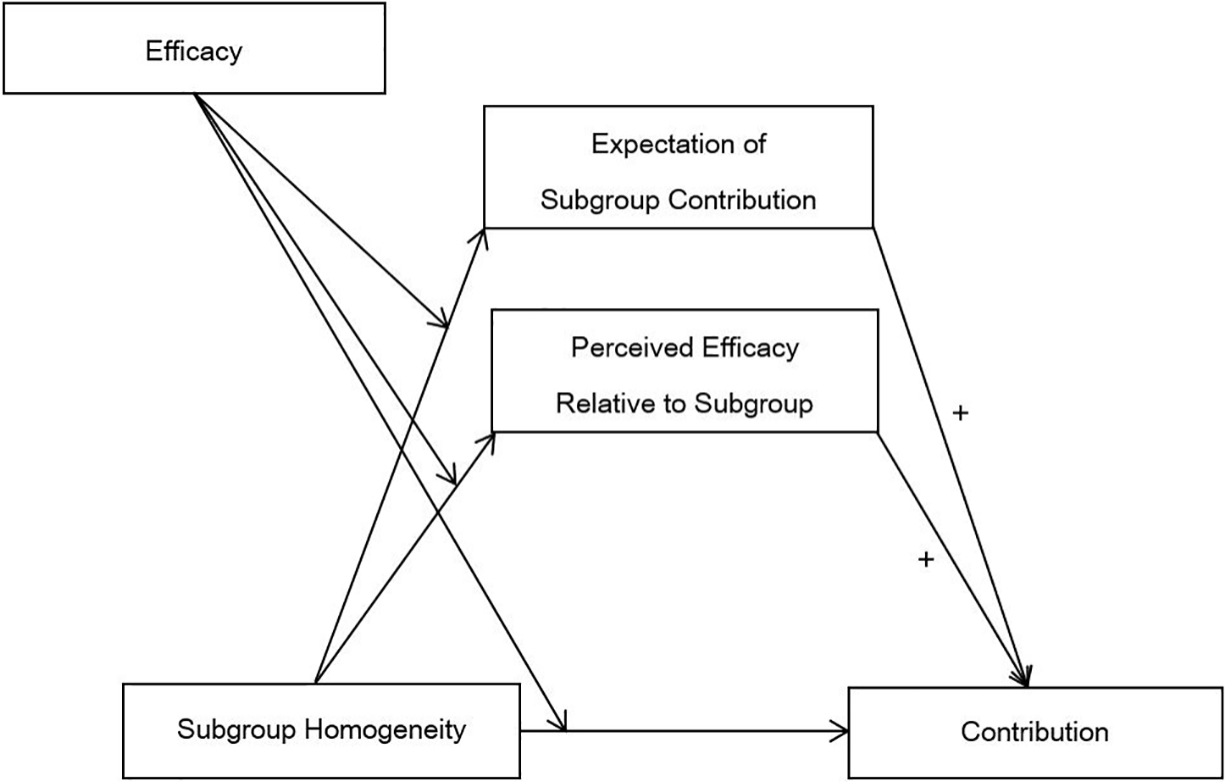
Proposed moderated mediation model stipulating how subgroup homogeneity influences contribution.

Taken together, we propose a moderated mediation model in which subgroup homogeneity and individual efficacy interact to affect contribution through two pathways, namely perceived efficacy of *oneself* relative to subgroup and expectation of subgroup contribution towards *others* (Fig 1).

## Method

### Participants

A total of 336 undergraduate students (63% female) participated in this study that was presented as an individual and group decision making experiment. Participants received a flat-rate of HK$50 (approximately US$6.5) for participation with a chance to earn an extra bonus according to their performance. The bonus ranged from HK$111 to HK$368 (US$14–47) with an average of HK$215 (US$28). This study was approved by the Survey and Behavioral Research Ethics Committee of The Chinese University of Hong Kong.

### Design

The experiment employed a 2 × 2 between-subject design with two levels of efficacy (high vs. low) and two levels of subgroup composition (homogenous vs. heterogeneous).

### Procedures

Six students participated in each session that lasted for around an hour. After participants gave their informed consent, the experimenter described the task scenario to them with visual aids. The PGD was presented as a social enterprise scenario. Participants role-played a member of a 12-person group in which group membership comprised of the participants from the present and the previous session. Their business was to produce cell phone straps using plastic beads. The completed cell phone straps could be sold through either participants’ own private stalls at a price of HK$5 per piece or the social enterprise at HK$15. The profit made by the social enterprise was a PG which would then be shared equally among all 12 participants. The total payoff for each participant was calculated as the sum of the profits earned from the private stall and the social enterprise.

Participants were then presented with four examples illustrating the characteristics of a PGD, such that everyone contributing a lot to the social enterprise is better than everyone contributing only a little; however, they could earn the most if they contribute only a little while others contribute a lot, and vice versa. The instructions stated clearly that there was no competition among participants and that there was also no competition between the social enterprise and private stalls. Participants’ first task was to manually produce these cell phone straps within a time limit. Their second task was to decide how many straps to sell through their own private stalls (non-contribution) and the social enterprise (contribution), respectively.

The experimenter then demonstrated how to make the cell phone straps using beads—by inserting beads into a plastic strap and then bending the end of the strap to fix the beads. Participants were given one minute as a practice trial to make the cell phone straps and then to make an allocation decision. Participants were also given a calculator to compute the profits of themselves and of other members based on a hypothetical scenario. The experimenter checked their answers to ensure that all of them understood the instructions clearly.

**Fig 2:**
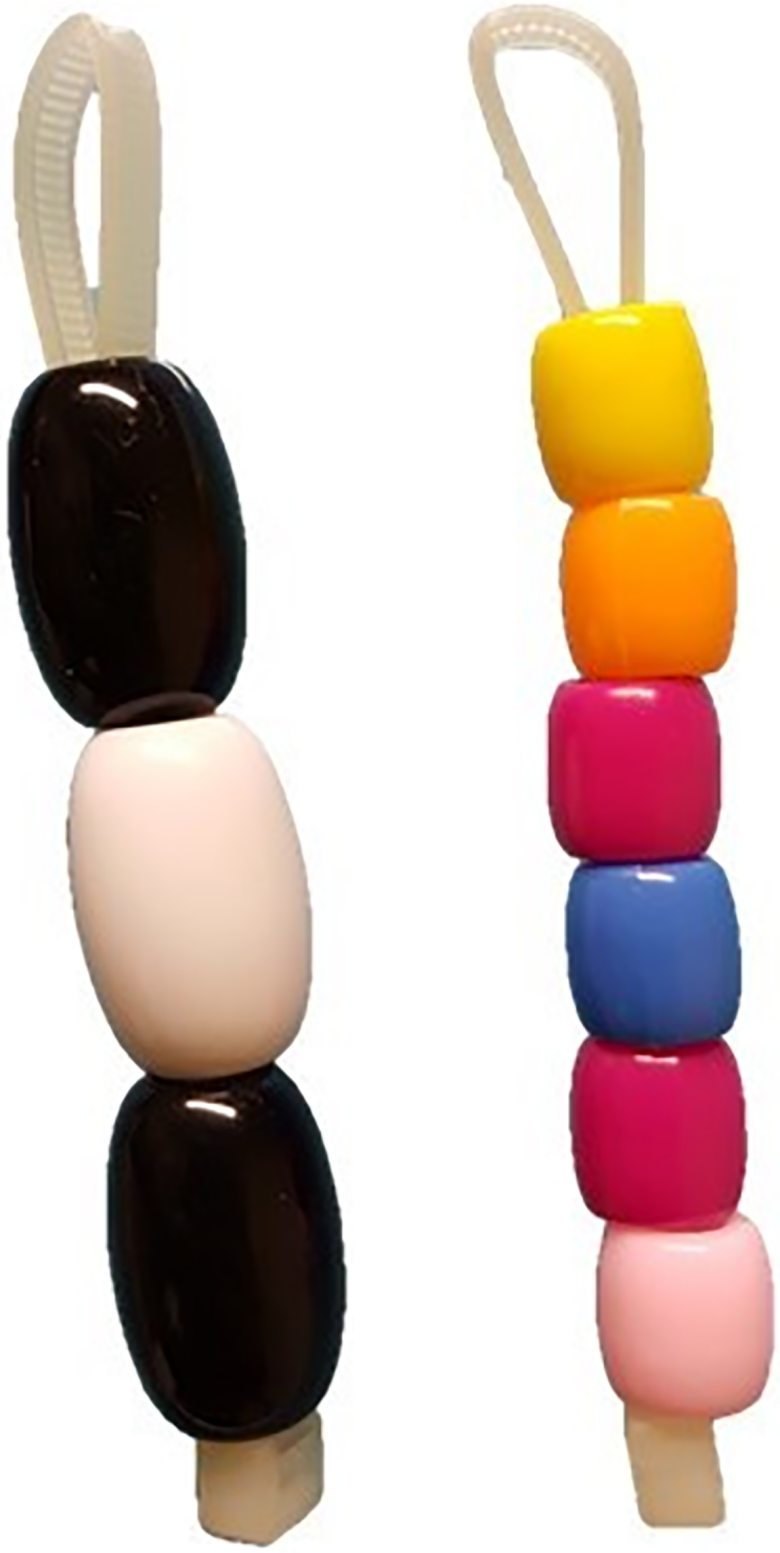
Example of the cell phone straps to be made by high-efficacy individuals and low-efficacy individuals respectively.

Afterwards, participants were told that as in everyday life, some people would have more time and they could work more efficiently. In order to simulate these individual differences, half of the 12 members in the social enterprise (i.e., the high-efficacy individuals) would be allotted more time (six minutes) and higher working efficiency (requiring only three large beads to produce a strap); whereas the other six members (i.e., the low-efficacy individuals) would be allotted less time (three minutes) and lower working efficiency (requiring six small beads to produce a strap) (Fig 2). S1 Appendix shows the instructions and illustrations of the task presented to the participants.

The six participants in each experimental session that constituted half of the 12-person social enterprise were assigned to two subgroups of three and were seated in two adjacent rooms. They were told that the 12-person group was allocated into two different sessions and each session was conducted in two separate rooms simply because of physical space constraints, in which we could not accommodate a 12-person group at the same physical location simultaneously (cf. Tajfel’s minimal group paradigm [49]). Inside each room, the three participants were seated face-to-face but were three meters apart from each other, such that they could not see others’ responses on the questionnaires. However, they could notice whether the other participants were given six or three minutes to produce the straps, and whether they had large or small beads. Because low-efficacy individuals had only three minutes to produce the cell phone straps, they were asked to complete an English-to-Chinese translation for a cell phone strap advertisement as a filler task while the high-efficacy individuals were still producing the cell phone straps in the remaining three minutes.

After participants had finished producing the cell phone straps within their allotted time, they completed a short questionnaire and allocated their straps to the social enterprise and their private stalls respectively by putting the straps into two separate compartments inside an opaque box that other people could not see their allocation decisions.

After the game, participants completed questionnaires concerning their experiences regarding the game. Full debriefing on the game was given to the participants upon completion of all experimental procedures. In order to motivate participants to make the allocation decisions seriously, one out of six participants from each session was randomly selected to receive a bonus which was equal to his or her earning in the game. They were told that the profit made by the social enterprise was calculated as the sum of the current and the previous session because the two sessions belonged to the same social enterprise. S2 Appendix describes how the bonus payments were computed.

### Manipulation of efficacy

Efficacy was manipulated in terms of both endowment (amount of time allotted to produce the cell phone straps) and efficiency (number of beads required to produce a strap) [29]. Participants were randomly assigned as either the low-efficacy or high-efficacy individuals. Low-efficacy individuals were given three minutes to produce the straps and six small beads were required to construct each strap; while high-efficacy individuals were given six minutes to produce the straps and each strap only required three large beads to construct.

### Manipulation of subgroup homogeneity

Although all social enterprises were identically comprised of six high-efficacy and six low-efficacy individuals, we systematically constructed subgroups of three people by assigning different numbers of high- and low-efficacy individuals to produce cell phone straps in separate rooms. There were two types of homogenous subgroups that consisted of either (a) three high-efficacy individuals or (b) three low-efficacy individuals; and two types of heterogeneous subgroups that consisted of either (a) two high- and one low-efficacy individuals or (b) one high- and two low-efficacy individuals.

### Psychological measures

After participants had finished making the cell phone straps, they reported their perceived efficacy and expectation of contribution before making the allocation decisions.

#### Perceived efficacy

Participants’ perceived efficacy relative to the subgroup was measured by the item “Compared to other members in the room, please estimate the amount of straps that you have made”, and participants’ perceived efficacy relative to the group was measured by the item “Compared to the other 11 members in the social enterprise, please estimate the amount of straps that you have made” on a 9-point scale with 1 = “The least in the group” and 9 = “The most in the group”.

#### Expectation of contribution

Participants also estimated the number of straps to be contributed by the other two members in the same room (expectation of subgroup contribution) and the other 11 members of the social enterprise (expectation of group contribution).

## Results

### Descriptive statistics

Table 2 shows the means and standard deviations of the number of straps produced, the number of straps contributed, and the proportion of straps contributed across different groups respectively. The average number of straps produced, average number of straps contributed, and average proportion of straps contributed by each individual were 23.3%, 10.0%, and 42.4%, respectively.

**Table 2:** Means (standard deviations) of the number of straps produced and contributed, and the proportion of straps contributed across different subgroup homogeneity and efficacy conditions (*N* = 336).

	Subgroup Homogeneity		
Efficacy	Homogeneous	Heterogeneous	Overall
Low	(*N* = 87)	(*N* = 81)	(*N* = 168)
Production	11.68 (1.63)	12.04 (1.56)	11.85 (1.60)
Contribution	4.60 (3.55)	5.16 (3.55)	4.87 (3.55)
Proportion	39.56% (30.20%)	43.05% (28.69%)	41.24% (29.45%)
			
High	(*N* = 87)	(*N* = 81)	(*N* = 168)
Production	34.52 (3.72)	35.10 (4.31)	34.80 (4.02)
Contribution	16.07 (9.17)	14.10 (10.20)	15.12 (9.70)
Proportion	46.77% (26.56%)	39.89% (28.26%)	43.45% (27.53%)
			
Overall	(*N* = 174)	(*N* = 162)	(*N* = 336)
Production	23.10 (11.81)	23.57 (12.01)	23.32 (11.89)
Contribution	10.33 (9.01)	9.63 (8.83)	9.99 (8.92)
Proportion	43.16% (28.59%)	41.47% (28.43%)	42.35% (28.48%)

*Note*. “Production” indicates the mean number of straps produced. “Contribution” indicates the mean number of straps contributed. “Proportion” indicates the *mean of individuals’* proportion of straps contributed.

### Effect of subgroup homogeneity on strap production and contribution

Before examining the effect of subgroup homogeneity on contribution, we tested whether the number of straps produced was different for individuals in homogeneous and heterogeneous subgroups. Welch’s t-test revealed no significant difference in strap production, *t*(331.38) = -0.36, *p* = .72. In order to provide more convincing evidence that individuals in homogeneous subgroups produced a similar number of straps as compared to that of heterogeneous subgroups, we followed the two one-sided tests (TOST) procedure proposed by Lakens [50] to conduct an equivalence test on strap production. We set the upper and lower equivalence bounds as Cohen’s *d* = ± .3. Power analysis showed that at least 145 participants per condition are required to achieve a power of .95 for this equivalence bound. Our sample size (*N* = 336) was adequate to perform this analysis. Using *α* = .05, the equivalence test was significant, *t*(331.38) = 2.39, *p* < .01, implying that the difference in strap production between homogeneous and heterogeneous subgroups was sufficiently close to zero to be considered practically equivalent [51].

Next, we tested the effect of subgroup homogeneity on strap contribution to the PG. Welch’s t-test revealed that there was no significant difference in strap contribution, *t*(333.10) = 0.72, *p* = .47. In order to test whether homogeneous subgroups contributed a similar number of straps as compared to that of heterogeneous subgroups, we also conducted the same equivalence test on strap contribution. The equivalence test on strap contribution was significant, *t*(333.10) = -2.03, *p* = .02, implying that homogeneous and heterogeneous subgroups contributed similar number of straps.

We also tested the effect of subgroup homogeneity on the proportion of straps contributed to the PG. Welch’s t-test revealed that there was no significant difference in proportion of straps contributed, *t*(332.54) = 0.55, *p* = .59. The equivalence test on proportion of straps contributed was significant, *t*(332.54) = -2.21, *p* = .01, implying that homogeneous and heterogeneous subgroups contributed similar proportion of straps.

In sum, individuals in homogeneous and heterogeneous subgroups produced similar number of straps, and contributed similar number and proportion of straps to the PG.

### Perceived efficacy and expectation of contribution as mediators

In order to test the proposed moderated mediation model stipulated in Fig 1, we performed a multilevel moderated mediation analysis [52] using Mplus 7.31 [53]. Multilevel modeling was employed because each low-efficacy or high-efficacy individual was nested within a homogeneous or heterogeneous subgroup. We assumed random effects in the model with efficacy as a level-1 predictor, subgroup homogeneity as a level-2 predictor, perceived efficacy relative to subgroup and expectation of subgroup contribution as two level-1 mediators, strap contribution as a level-1 dependent variable, and subgroup number as a cluster variable. The parameters in the model were estimated using maximum likelihood (ML). Three responses were excluded from this moderated mediation analysis because the participants did not indicate their expectation of subgroup contribution. S3 Appendix shows the Mplus code of this analysis.

**Fig 3:**
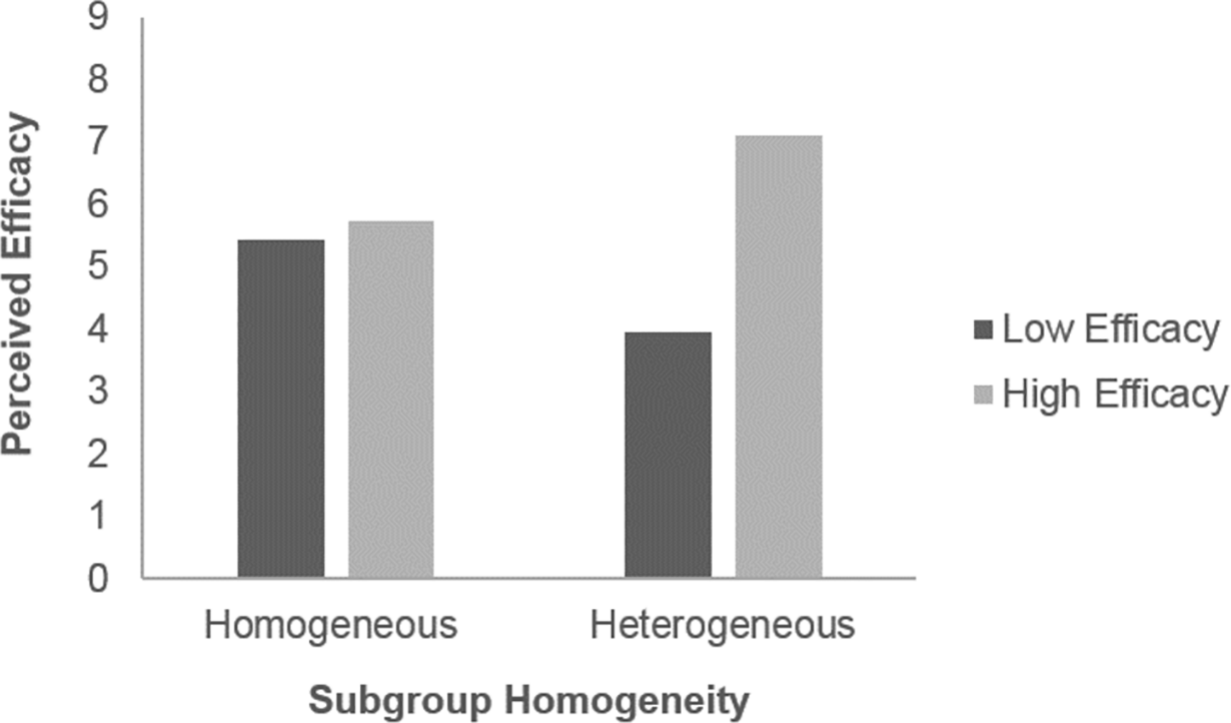
Perceived efficacy relative to subgroup across different conditions.

Hypothesis 1 states that absolute efficacy moderates the effect of subgroup homogeneity on perceived efficacy, such that high-efficacy individuals in heterogeneous subgroups will have a higher perceived efficacy than those in homogeneous subgroups; whereas low-efficacy individuals in homogeneous subgroups will have a higher perceived efficacy than those in heterogeneous subgroups. Supporting Hypothesis 1, we found that the effect of subgroup homogeneity on perceived efficacy relative to subgroup was significantly different for high-efficacy and low-efficacy individuals, *b* = 3.19, *p* < .001, 95% CI [2.24, 4.13]. High-efficacy individuals in heterogeneous subgroups had higher perceived efficacy than those in homogeneous subgroups, *b* = 1.52, *p* < .001, 95% CI [0.93, 2.10]; low-efficacy individuals in homogeneous subgroups had higher perceived efficacy than those in heterogeneous subgroups, *b* = -1.67, *p* < .001, 95% CI [-2.41, -0.94] (Fig 3). Hypothesis 1 further states that there would be a moderated mediation effect in which subgroup homogeneity and efficacy interact to affect perceived efficacy relative to subgroup, which in turn mediate the effect of subgroup homogeneity on contribution. For high-efficacy individuals, the conditional indirect effect of subgroup homogeneity on strap contribution through perceived efficacy as a mediator was significant, *b* = 1.13, *p* < .01, 95% CI [0.35, 1.91], such that high-efficacy individuals in heterogeneous subgroups had higher perceived efficacy and hence contributed more straps. For low-efficacy individuals, the conditional indirect effect was also significant, *b* = -1.24, *p* = .01, 95% CI [-2.19, -0.30], such that low-efficacy individuals in homogeneous subgroups had higher perceived efficacy and hence contributed more straps. The index of moderated mediation [54] was significant, *b* = 2.37, *p* < .01, 95% CI [0.79, 3.95]. These results supported Hypothesis 1 that perceived efficacy was a mediator of the effect of subgroup homogeneity on strap contribution, and that the mediating effects were in opposite directions for high-efficacy and low-efficacy individuals.

**Fig 4:**
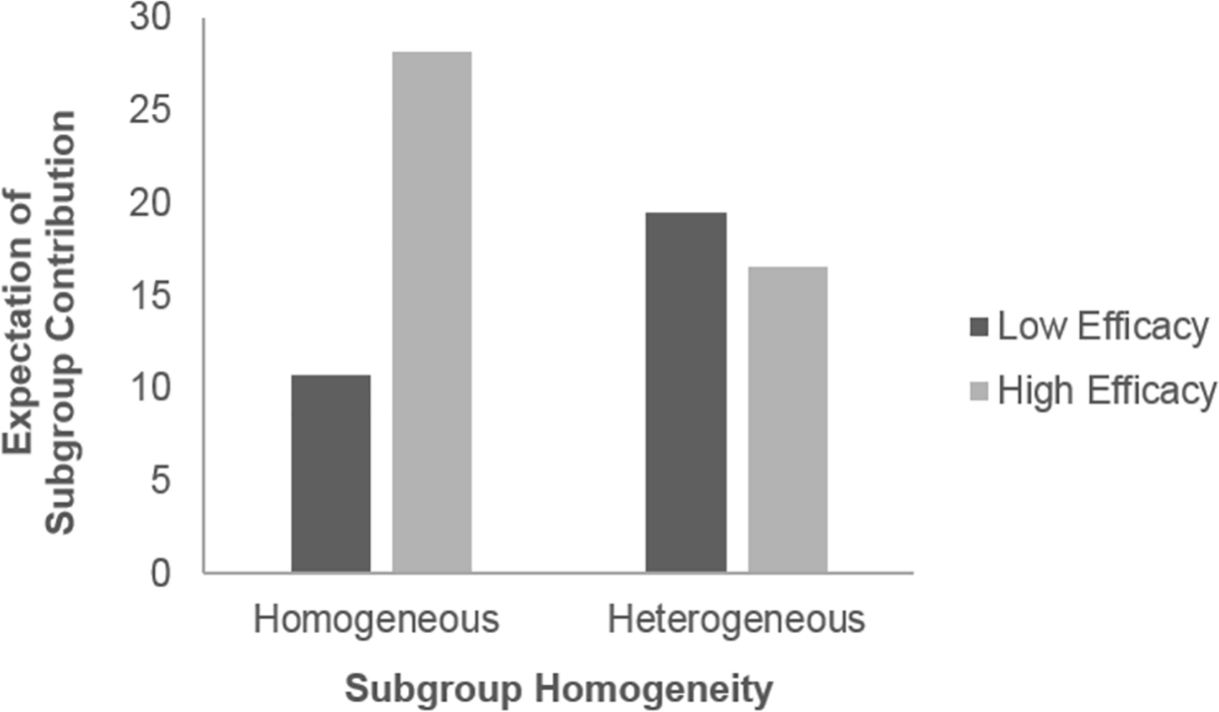
Expectation of subgroup contribution across different conditions.

Hypothesis 2 states that absolute efficacy moderates the effect of subgroup homogeneity on expectation of subgroup contribution, such that high-efficacy individuals in homogeneous subgroups will have a higher expectation of subgroup contribution than those in heterogeneous subgroups; whereas low-efficacy individuals in heterogeneous subgroups will have a higher expectation of subgroup contribution than those in homogeneous subgroups. Supporting Hypothesis 2, we found that the effect of subgroup homogeneity on expectation of subgroup contribution was significantly different for high-efficacy and low-efficacy individuals, *b* = -21.76, *p* < .001, 95% CI [-27.30, -16.22]. High-efficacy individuals in homogeneous subgroups had a higher expectation of subgroup contribution than those in heterogeneous subgroups, *b* = -12.83, *p* < .001, 95% CI [-16.98, -8.69]; low-efficacy individuals in heterogeneous subgroups had a higher expectation of subgroup contribution than those in homogeneous subgroups, *b* = 8.92, *p* < .001, 95% CI [4.82, 13.03] (Fig 4). Hypothesis 2 further states that there would be a moderated mediation effect in which subgroup homogeneity and efficacy interact to affect expectation of subgroup contribution, which in turn mediates the effect of subgroup homogeneity on contribution. For high-efficacy individuals, the conditional indirect effect of subgroup homogeneity on strap contribution through expectation of subgroup contribution as a mediator was significant, *b* = -4.32, *p* < .001, 95% CI [-6.33, -2.31], such that high-efficacy individuals in homogeneous subgroups had higher expectation and hence contributed more straps. For low-efficacy individuals, the conditional indirect effect was also significant, *b* = 3.00, *p* < .001, 95% CI [1.37, 4.64], such that low-efficacy individuals in heterogeneous subgroups had higher expectation and hence contributed more straps. The index of moderated mediation was significant, *b* = -7.33, *p* < .001, 95% CI [-10.31, -4.34]. These results supported Hypothesis 2 that the expectation of subgroup contribution was a mediator of the effect of subgroup homogeneity on strap contribution, and that the mediating effects were in opposite directions for high-efficacy and low-efficacy individuals.

As an exploratory analysis, we tested a similar moderated mediation model in which the two mediators (i.e., perceived efficacy relative to the *subgroup* and expectation of *subgroup* contribution) were substituted by perceived efficacy relative to the *group* and expectation of *group* contribution, respectively. None of the moderated mediation effects were significant. These null results further demonstrated that it was the subgroup-level variables but not the group-level variables that affected the contributions to the group.

## Discussion

This study investigated whether we can maximize contribution through manipulating subgroup homogeneity in PGD that the whole group shared the same PG. Our results suggested that splitting group members into homogeneous or heterogeneous subgroups can concurrently enhance and reduce contribution through two different mechanisms. Consider the high-efficacy individuals in homogeneous subgroups, they had a lower perceived efficacy relative to subgroup than those in heterogeneous subgroups, which led them to contribute less. However, they had higher expectation of subgroup contribution than those in heterogeneous subgroups, which led them to contribute more. The effects of perceived efficacy and expectation of contribution hence counteracted each other and resulted in similar amounts of contribution for high-efficacy individuals. Similarly, low-efficacy individuals in homogeneous subgroups had a higher perceived efficacy, but lower expectation of contribution than those in heterogeneous subgroups. The effects of perceived efficacy and expectation of contribution again counteracted each other and resulted in similar amounts of contribution for low-efficacy individuals. Hence, it appears that perceived efficacy and expectation of cooperation nullified each other, helping to explain why homogeneous subgroups contributed to the same extent as heterogenous subgroups did.

In an organization, it is common to divide workers into different subgroups to work on a specific task. Oftentimes, members of a subgroup have different levels of efficacy. Our results suggest that different strategies may be needed to motivate high- and low-efficacy workers to contribute to the organization. High-efficacy workers, who supposedly have a higher perceived efficacy, may expect other low-efficacy workers to contribute only a little, resulting in their reluctance to contribute more. Hence, interventions should focus on changing their expectation of the contribution of other members, for instance, by convincing them that all members, regardless of their levels of efficacy, are equally committed to the organization. On the contrary, low-efficacy workers may expect other workers to contribute considerably more, meaning that they may see themselves to be less critical to the organization. Hence, interventions should focus on increasing their perceived efficacy, for instance, by emphasizing that their strengths and uniqueness are valuable to and have critical impacts on the organization.

The negligible difference between groups composing of homogeneous subgroups and groups composing of heterogeneous subgroups should not be surprising after all. Research in work group diversity has long been reporting mixed findings, e.g., [19, 55]. On the one hand, a social categorization or identity related perspective would advocate that homogeneous groups are better because having members of similar characteristics promotes a positive affective state such as stronger group identity, cohesiveness, and trust. On the other hand, an information processing or decision-making perspective argues that heterogeneous groups are better because members with diverse backgrounds promotes positive cognitive state of differences in knowledge and expertise, resulting in better decision making. Our work nicely demonstrates that different emergent states like perceived efficacy and expectation of cooperation can nullify the effects of each other as a result of the interaction between group diversity and member characteristics (i.e., subgroup configuration and absolute efficacy in the current study).

In this experiment, participants made their contribution decision based on their number of straps produced. This effort task–manufacturing cell phone straps–to determine their endowment was similar to other public good experiments that examined the “house money effect”: comparing endowments “given” by the experimenter versus endowments “earned” by the players. These experiments determined endowments by asking participants to crack walnuts [56], answer Graduate Management Aptitude Test questions, e.g., [57], or stuff letters into envelops [58]. We anticipated that through random assignment of participants to experimental conditions, heterogeneity in endowments (due to differences in ability in manufacturing cell phone straps) would be unsystematic random errors that affected both homogeneous and heterogenous subgroups similarly. We understand that this “earned endowment” manipulation adds another layer of heterogeneity. However, we believe that the heterogeneity arising from the “earned endowment” manipulation is not a confounding factor but instead it provides ecological validity; unlike other experiments in which endowments were fixed. Nonetheless, we examined how the effect of subgroup homogeneity on strap contribution could be confounded by strap production. We found that individuals in homogeneous subgroups produced similar number of straps as compared to those in heterogeneous subgroups. Furthermore, the effect of subgroup homogeneity on strap contribution remained non-significant even after controlling for strap production, *F*(1, 333) = 1.36, *p* = .24. We can therefore rule out the possibility that one type of subgroups may have produced more straps than another type of subgroups that have affected strap contribution.

Due to physical space limitations, we conducted each experimental session with six participants only instead of twelve participants, which was the group size of the social enterprise. However, we reiterated various times during the experiment that the social enterprise was comprised of 12 participants. In addition, written descriptions of the task scenario presented in a large font size posted on the wall, which also stated that their group consisted of six high-efficacy and six low-efficacy members, were clearly visible to the participants throughout the experiment. During the practice trial, we also asked participants to make estimates and computations regarding the other 11 participants in their group. Their answers to the practice exercise were checked by the experimenter and further explanation was given to clarify any misunderstandings. We are confident that participants understood clearly that it was the 12-person group, but not the 3-person group in their room nor the 6-person group in that session, that shared the PG.

There are several limitations in our study. Our moderated mediation analysis identified perceived efficacy relative to subgroup and expectation of subgroup contribution as two mediator variables. However, we are aware that such a “measurement-of-mediation” design does not imply causation between the mediators and the dependent variable [59]. Particularly, one may argue that the cause-and-effect relation between expectation of contribution and contribution may be unclear, because it is also possible that an individual who chooses to contribute a lot may be susceptible to a false consensus bias that others will also contribute a lot [60]. We attempted to minimize this effect by measuring the participants’ expectation *before* they made a decision on contribution. Furthermore, this effect presumably would not influence the interaction effects between efficacy and subgroup homogeneity, such that it should not have posed a significant impact on the validity of our results.

While homogeneous groups are conceptually simple and unambiguous in the sense that all members have the same efficacy, our treatment of heterogeneity, however, could have been overly simplified. The concept of group heterogeneity is complex because there are numerous qualitatively different distributions of group composition. Our heterogeneous subgroups belonged to the type of a “minority belief” distribution according to DeRue et al. [61]. It is difficult to assert how our findings may be generalized to other types of heterogeneous distributions, for instances, the bimodal distribution (equal number of high- and low-efficacy members), the fragmented distribution (all members having different efficacy [61]), or even a hegemony distribution [24]. Future research shall examine the robustness of our results across different subgroup compositions.

Past findings about the effect of group homogeneity of efficacy on contribution have been mixed. Our study showed that perceived efficacy and expectation of contribution are two mechanisms that can counteract the effect of homogeneity of efficacy on contribution. It is possible that certain factors in past experimental designs, including the presentation methods of PGD scenarios, the existence of a provision threshold, the group size, and the payoff structure, etc., can make either the effect of perceived efficacy or expectation of contribution more salient, which in turn causes either the homogeneous or heterogeneous group to contribute more. Because past studies did not measure the perceived efficacy and expectation of contribution of the participants, future research can fill in the gap by investigating how these factors may influence the effect of homogeneity of efficacy on contribution and their respective mechanisms.

While we are cautious not to over-generalize our findings theoretically and empirically because of our relatively narrow operationalization of group heterogeneity and the specific subgrouping manipulation that was based on isolation in physical space, our findings are useful in showing how subgrouping according to efficacy levels of individuals can have both advantageous and detrimental effects at the same time on contribution in mixed-motive situations.

## Supporting information

S1 Appendix Instructions and illustrations of the experimental task. (PDF)

S2 Appendix Computation of bonus payments. (DOCX)

S3 Appendix Mplus code of the multilevel moderated mediation model. (DOCX)

S4 Appendix Additional data analyses on subgroup configurations. (DOCX)

S1 Raw Data Raw data of the reported study containing variables relevant to this paper. (SAV)
