# Supplementary material for: The effect of subgroup homogeneity of efficacy on contribution in public good dilemmas
Source: PLoS One. 2018 Jul 31;13(7):e0201473. doi: 10.1371/journal.pone.0201473 (PMC6067760; doi:10.1371/journal.pone.0201473)
Supplement: S1 Appendix — (PDF) [file pone.0201473.s001.pdf]

Original Instructions in Chinese

(English version follows Chinese version)

The effect of subgroup homogeneity of efficacy on contribution in public good dilemmas

Paton Pak-chun Yam1, Gary Ting Tat Ng2, Wing Tung Au2*, Lin Tao3, Su Lu4, Hildie Leung5, Jane M. Y. Fung2

1 Department of Psychological and Behavioural Science, London School of Economics and Political Science

2 Department of Psychology, The Chinese University of Hong Kong, Hong Kong

3 Department of Sociology, Peking University, Beijing, China

4 Department of Human Resource and Organizational Behavior, University of International Business and Economics, Beijing, China

5 Department of Applied Social Sciences, The Hong Kong Polytechnic University, Hong Kong

實驗程序簡介

- 簽署實驗同意書
- 簡介實驗處境及活動規則
- 活動練習
- 進行實驗
- 回答問卷
- 抽獎及派錢

電話繩小生意

- 出售途徑:

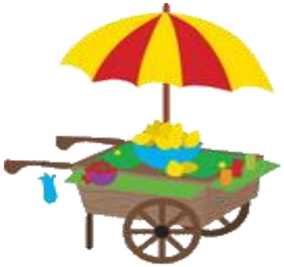

1. 個人攤檔
   - 跳蚤市場檔位售賣。
   - 所有利潤將全數歸於自己。

或者

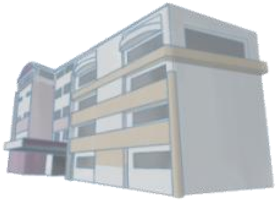

2. 社會企業
   - 社區中心轄下的社會企業售賣。
   - 社企由12 個 會員組成(包括自己) 。
   - 社企會收集各會員的電話繩，然後以企業的名義作公開出售。
   - 社企得到的總收益將分給所有會員。

- 個人攤檔和社會企業的收益是**<u>有分別</u>**的。

從**個人攤檔**得到的收入

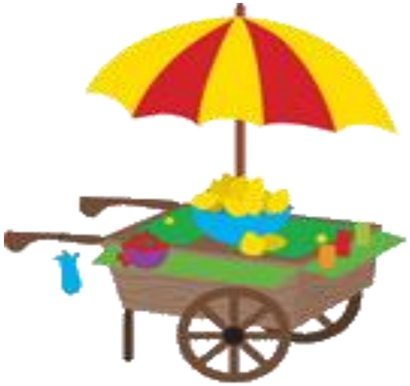

售價：

　$5 / 每條

收入：

　$5 * (你放在個人攤檔的電話繩數量)

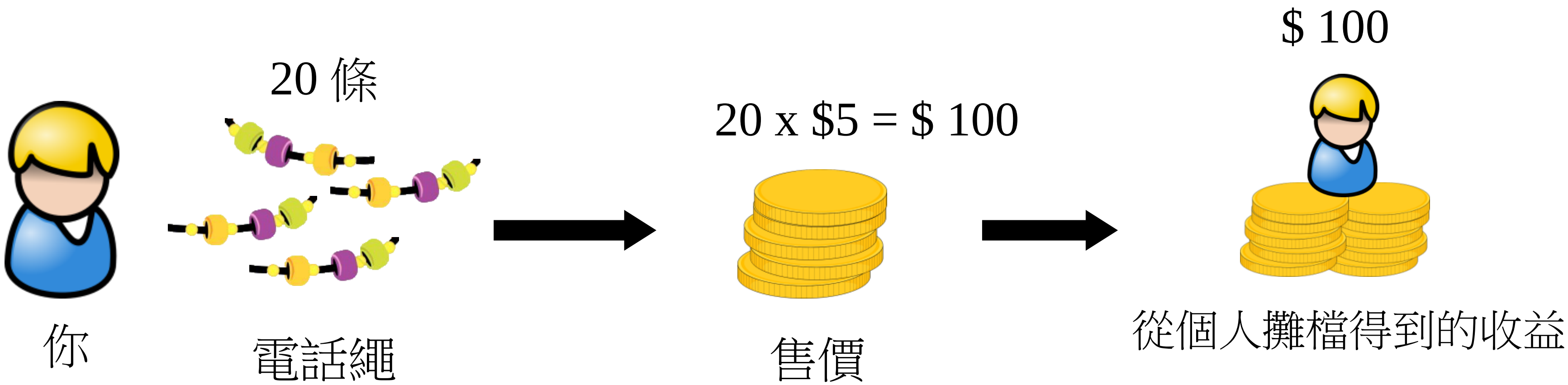

從**社會企業**得到的收入

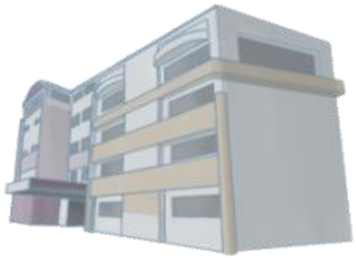

售價:

$15 / 每條

收入:

$$\frac{\text{(所有會員給予社企電話繩的總數量) * \$15}}{\text{12位會員}}$$

其他11位會員

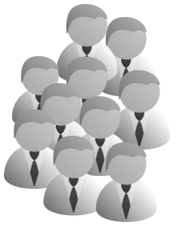

電話繩: 130 條

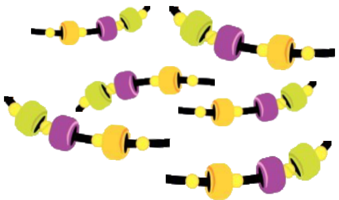

你

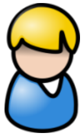

電話繩: 20條

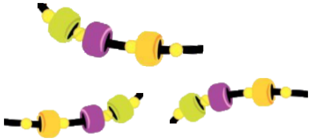

社企共收得

130 + 20= 150條電話繩

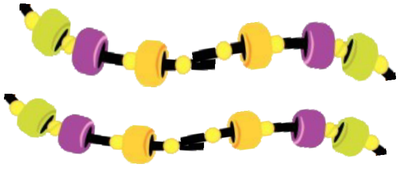

(150 * $15) ÷ 48

~ $188

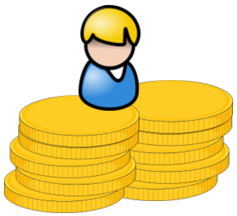

從社會企業得到的收益

社會企業

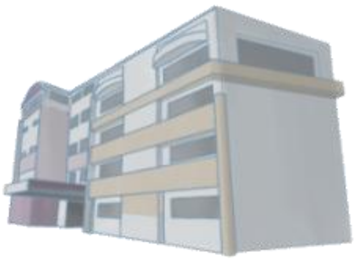

A. 由於社企的知名度比個人攤檔的知名度高，所以電話繩所賣得的收益會比經由個人攤檔所賣得的收益為多。

B. 每一個會員，不論把多少條電話繩放在社企，甚至沒有把任何電話繩放在社企，都會分到相同的利潤。

C. 你可能比其他會員給予社企較多或較少電話繩，但每會員收到的利潤<u>都是一樣</u>。

目標: 獲得最高的利潤

- **你的總收入 = 個人攤檔之收益 + 社會企業分發之收益**
- 把愈多的電話繩放在個人攤檔，可分配到社企的電話繩便愈少。
- 你要決定把多少條電話繩放在個人攤檔及社會企業。

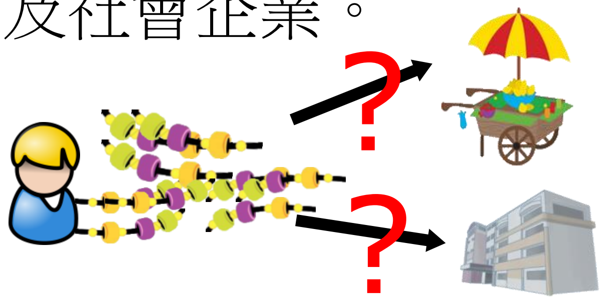

- 各會員之間並不存在任何競爭。
- 個人檔攤和社會企業之間不存在任何競爭。
- 社企收到電話繩愈多，各會員從社企收到的利潤亦愈多

例子 1

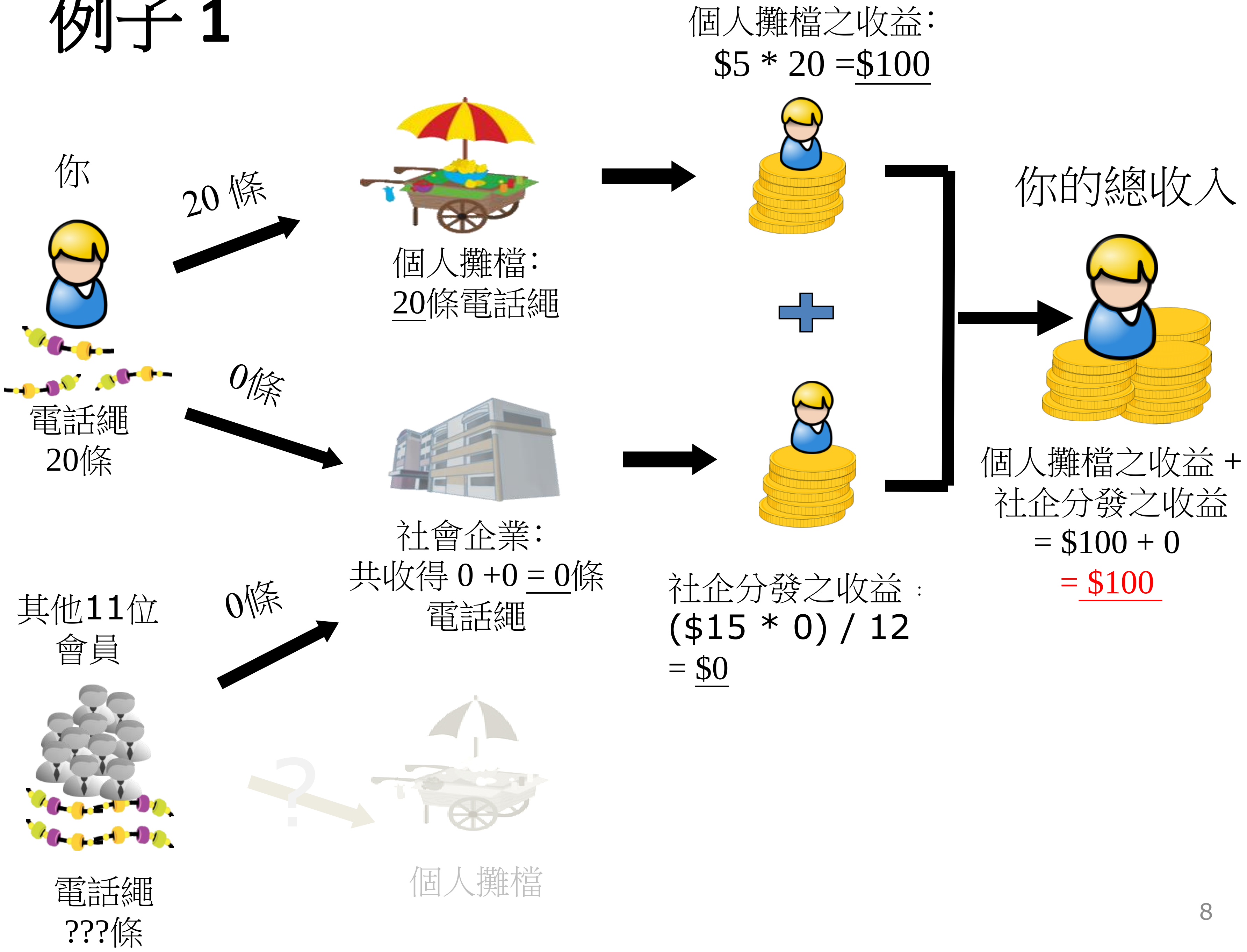

個人攤檔之收益:
$5 * 20 =$100
你
20 條
個人攤檔:
20條電話繩
你的總收入
電話繩
20條
0條
社會企業:
共收得 0 +0 = 0條
電話繩
個人攤檔之收益 +
社企分發之收益
= $100 + 0
= $100
其他11位
會員
0條
社企分發之收益 :
($15 * 0) / 12
= $0
?
個人攤檔
電話繩
???條

例子 2

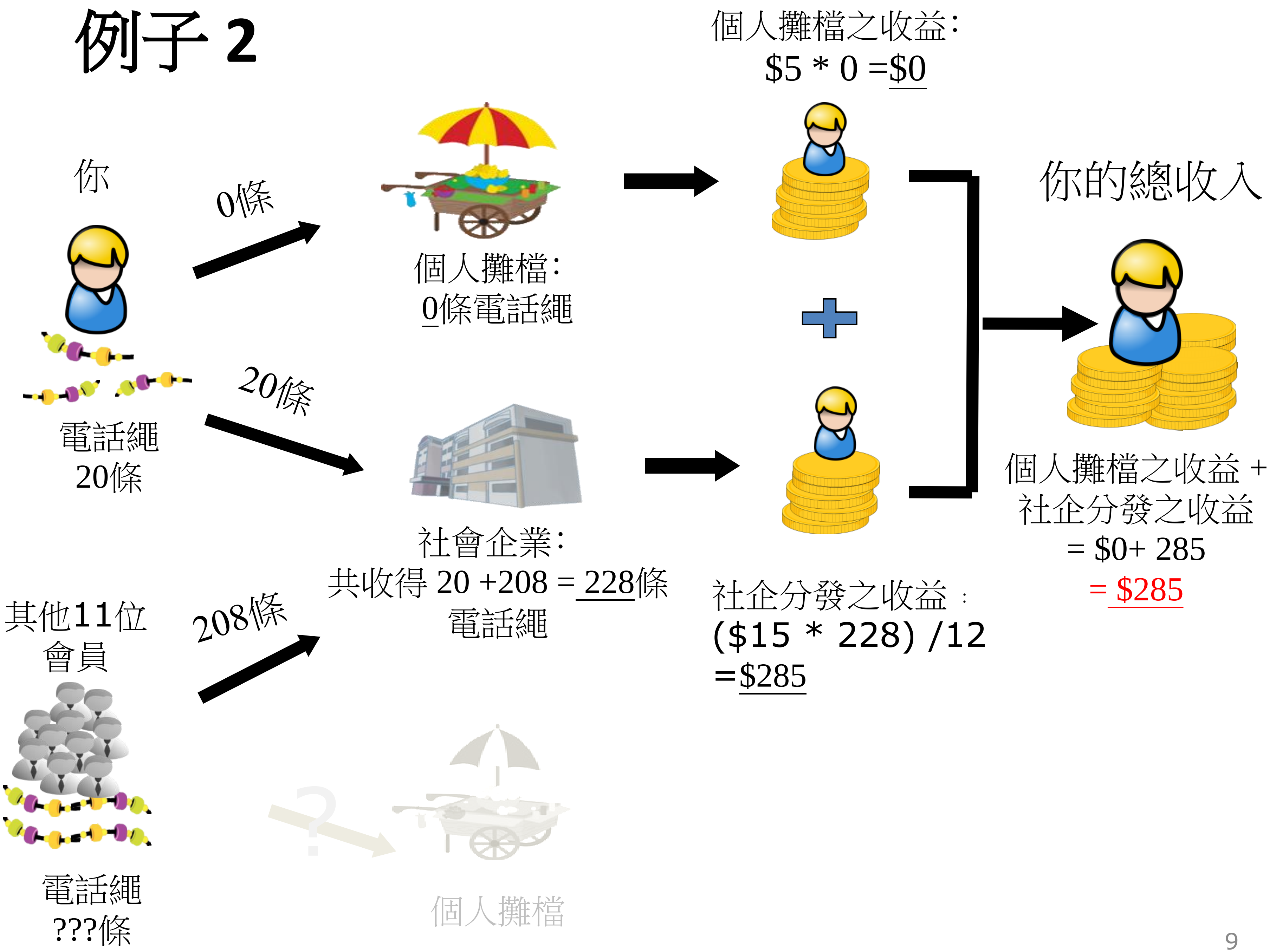
個人攤檔之收益:
$5 * 0 =$0
你
0條
個人攤檔:
0條電話繩
你的總收入
電話繩
20條
20條
社會企業:
共收得 20 +208 = 228條
電話繩
個人攤檔之收益 +
社企分發之收益
= $0+ 285
= $285
社企分發之收益：
($15 * 228) /12
=$285
其他11位
會員
208條
?
電話繩
???條
個人攤檔

例子 3

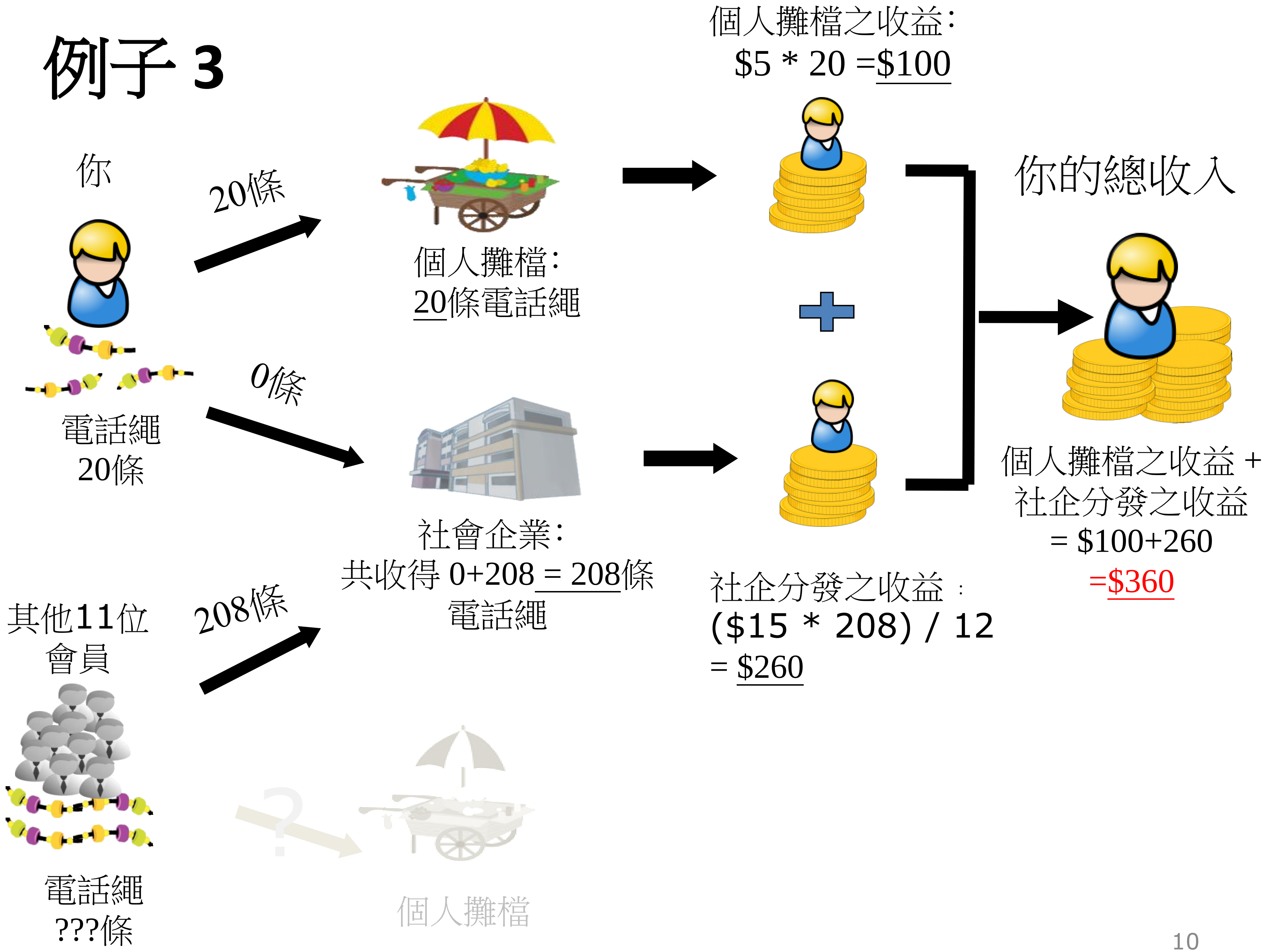

你
電話繩
20條
20條
0條
個人攤檔:
20條電話繩
社會企業:
共收得 0+208 = 208條
電話繩
其他11位
會員
電話繩
???條
208條
個人攤檔
個人攤檔之收益:
$5 * 20 =$100
社企分發之收益：
($15 * 208) / 12
= $260
你的總收入
個人攤檔之收益 +
社企分發之收益
= $100+260
=$360

例子 4

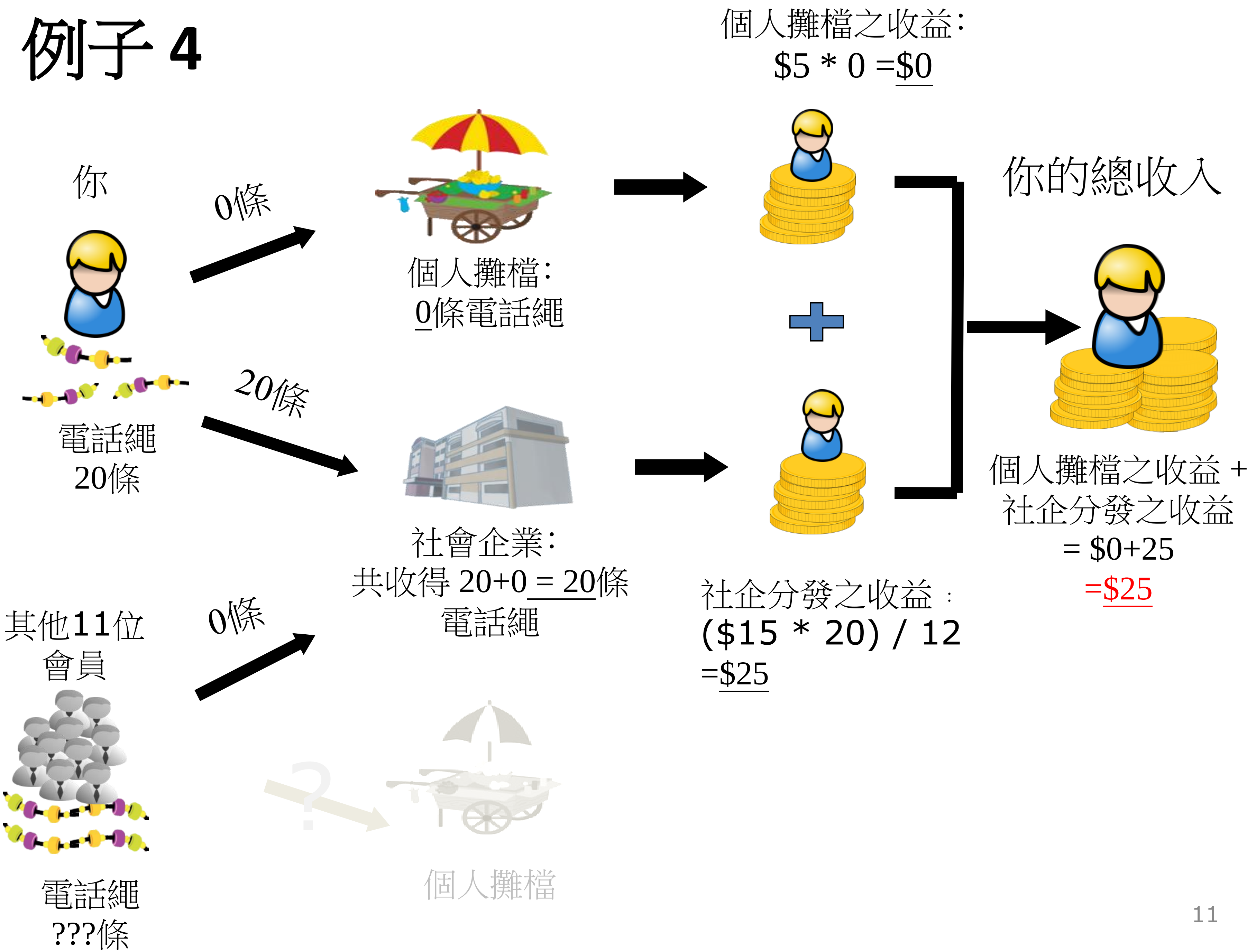
個人攤檔之收益：
$5 * 0 =$0
你
0條
個人攤檔：
0條電話繩
你的總收入
電話繩
20條
20條
社會企業：
共收得 20+0 = 20條
電話繩
個人攤檔之收益 +
社企分發之收益
= $0+25
=$25
其他11位
會員
0條
社企分發之收益：
($15 * 20) / 12
=$25
?
個人攤檔
電話繩
???條

製作電話繩**規則**

- 社企一半 會員 :: 大珠子及6分鐘

  另一半會員 :: 小珠子及3分鐘

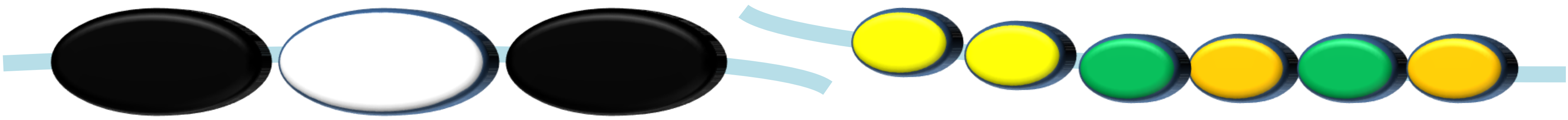

- 少時間的人會在餘下的3 分鐘完成一些翻譯練習

- 完成的電話繩，放到透明盒子裡

- 目標: (1) **製作最多的電話繩**

  (2) 分配電話繩到個人攤檔及社會企業以**獲得最高的利潤**

Translated Instructions in English

Overview of Experimental Procedure

- Sign the informed consent
- Instructions of the experiment
- Practice trials
- Conduct the experiment
- Fill in the questionnaires
- Lucky draw and payment

Running a small business in phone straps

- Retail channels:

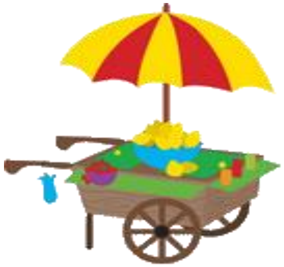

1. Individual stalls

- Sold at flea market stalls
- All the income belongs to yourself

OR

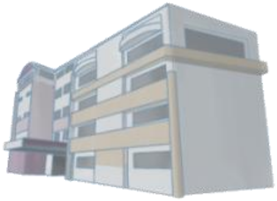

2. Social enterprise

- Sold by a social enterprise in a community center
- The social enterprise is formed by 12 members (including yourself)
- The social enterprise collects the phone straps made by its members, and resells them publicly under the name of the enterprise
- The social enterprise distributes the total income among all the members

- The income received from the individual stalls and that from the social enterprise are **DIFFERENT**

Income from the **individual stalls**

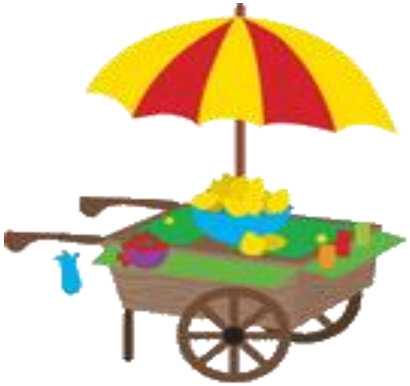

Selling price :

$5 per piece

Income :

$5 x (number of phone straps that you sell at the stall)

$ 100

20 pieces

20 x $5 = $ 100

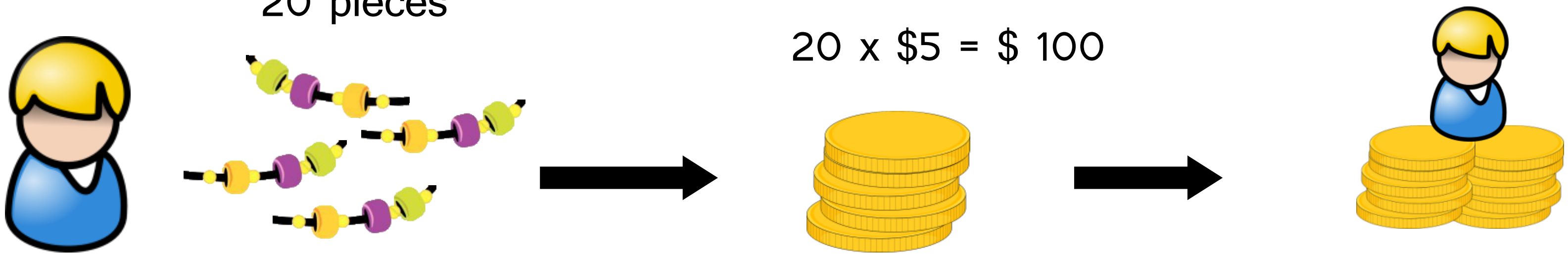

You

Phone straps

Price

Income from the individual stall

Income from the **social enterprise**

Selling price ：

$15 per piece

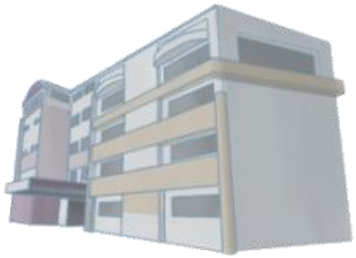

Income ：

$$\frac{(\text{total number of phone straps given by the members}) \times \$15}{12 \text{ members}}$$

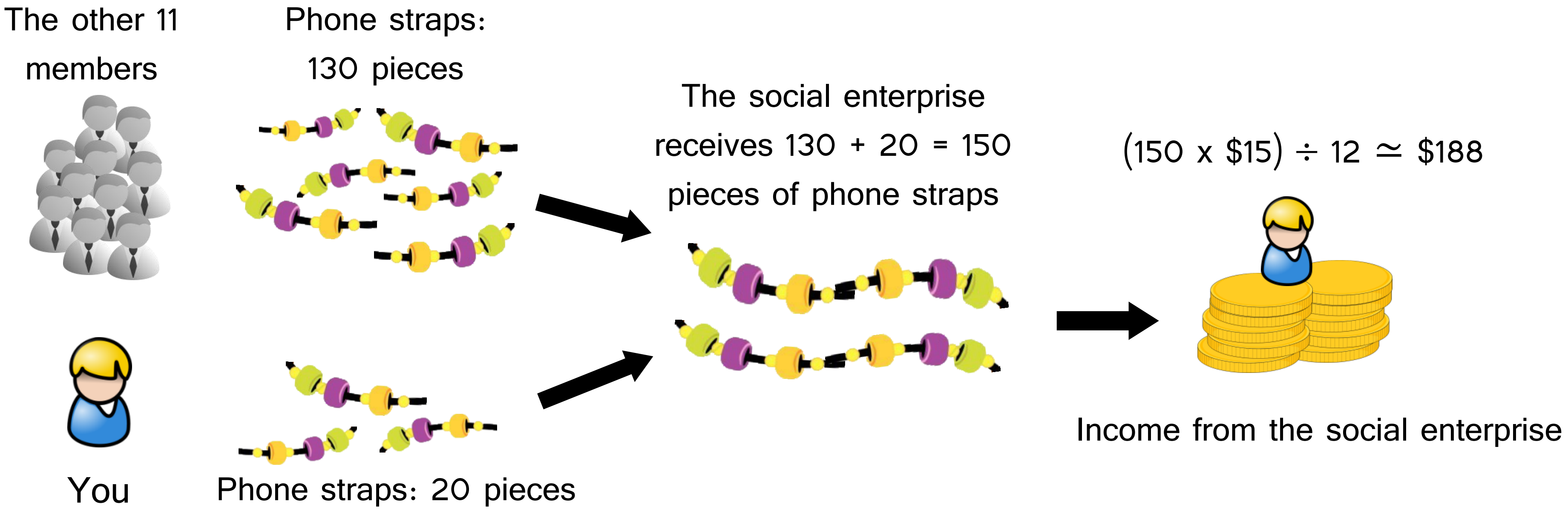

Social enterprise

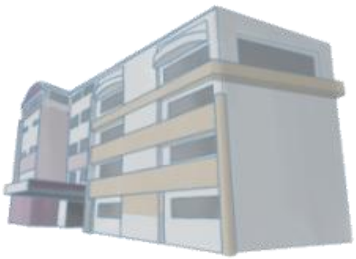

A. Since the social enterprise is more well-known than the individual stalls, its income generated from selling phone straps will be higher than that of its counterpart.

B. Regardless of the number of phone straps given to the social enterprise, every member will always receive the same amount of profit as distributed by the enterprise, even if he or she has not given any phone straps to the enterprise at all.

C. As compared to the other 11 members, you may have given more (or less) phone straps to the social enterprise, yet everyone will always share the same amount of profit from the enterprise.

Goal: **Obtain the highest profit**

- **Your total income =**
  Income from the individual stall + Income from the social enterprise

- The more phone straps you sell at the individual stall, the less you can allocate to the social enterprise

- You need to decide on how many phone straps to be allocated to the individual stall and the social enterprise, respectively

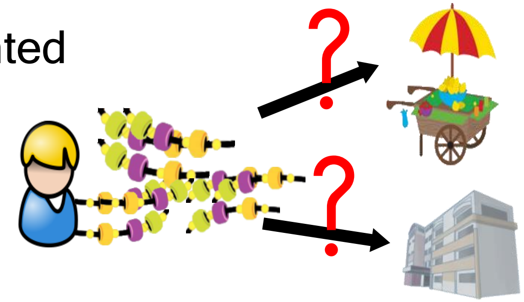

- There does not exist any competition among the members

- There does not exist any competition between the individual stalls and the social enterprise

- The more phone straps that the social enterprise receives, the more profit that the members receive from the enterprise

Example 1

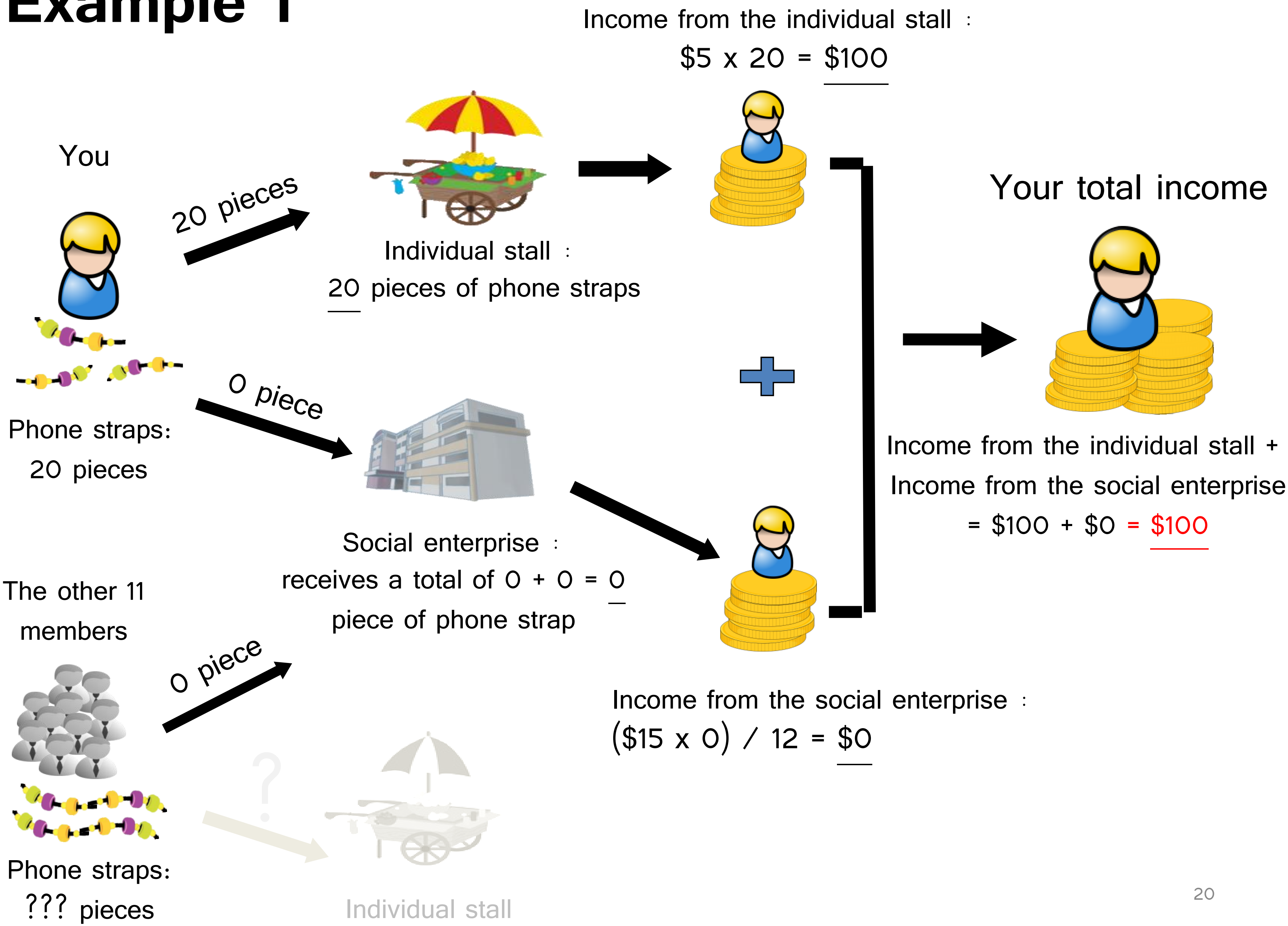
Income from the individual stall :
$5 x 20 = $100
You
20 pieces
Individual stall :
20 pieces of phone straps
Your total income
Phone straps:
20 pieces
0 piece
Income from the individual stall +
Income from the social enterprise
= $100 + $0 = $100
Social enterprise :
receives a total of 0 + 0 = 0
piece of phone strap
The other 11
members
0 piece
Income from the social enterprise :
($15 x 0) / 12 = $0
Phone straps:
??? pieces
Individual stall

Example 2

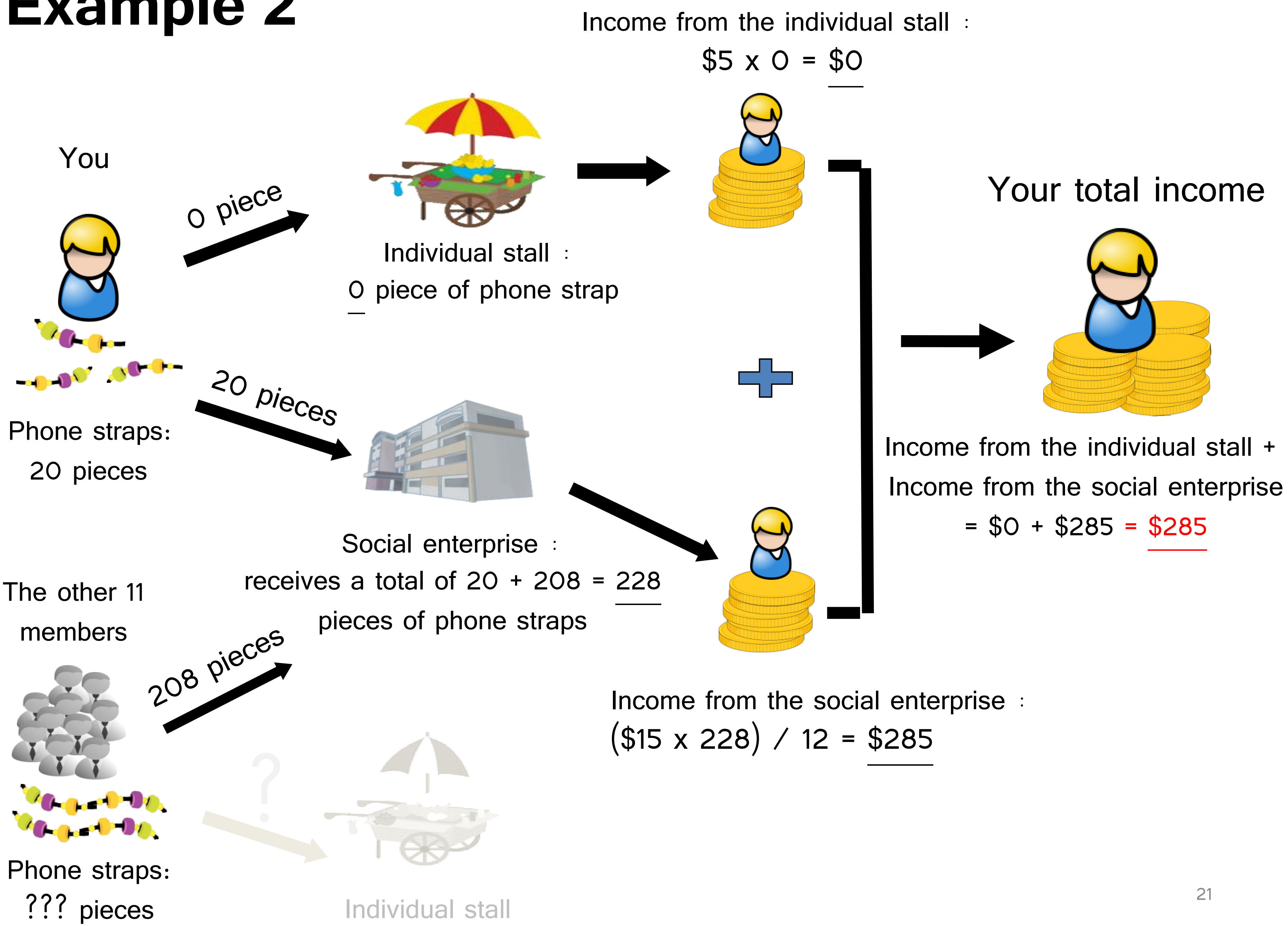

Income from the individual stall ：
$5 x 0 = $0
You
0 piece
Individual stall ：
0 piece of phone strap
Phone straps:
20 pieces
20 pieces
Your total income
Income from the individual stall +
Income from the social enterprise
= $0 + $285 = $285
Social enterprise ：
receives a total of 20 + 208 = 228
pieces of phone straps
The other 11
members
208 pieces
Income from the social enterprise ：
($15 x 228) / 12 = $285
Phone straps:
??? pieces
Individual stall

Example 3

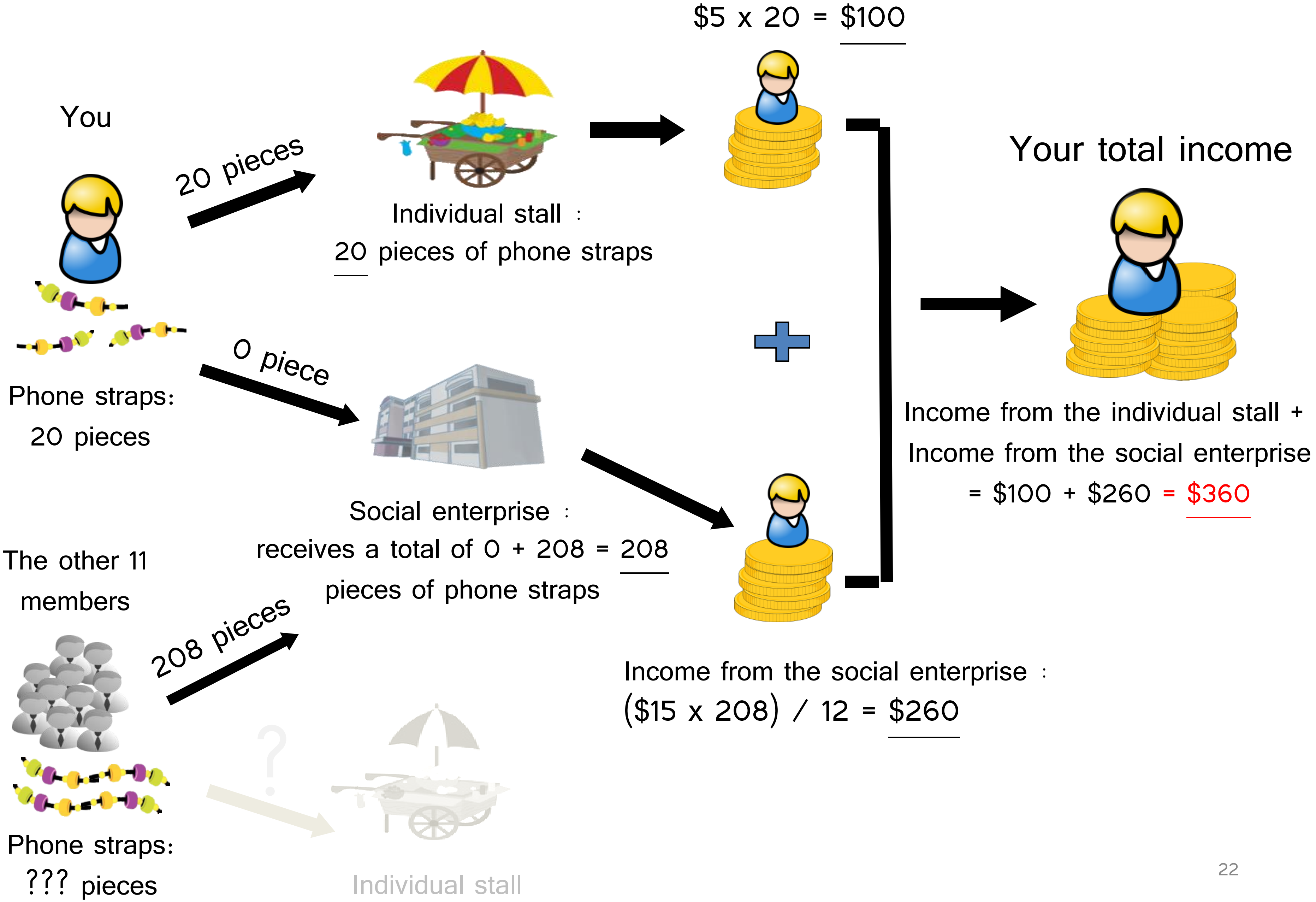
You
20 pieces
Individual stall :
20 pieces of phone straps
Income from the individual stall :
$5 x 20 = $100
Phone straps:
20 pieces
0 piece
Social enterprise :
receives a total of 0 + 208 = 208
pieces of phone straps
The other 11
members
208 pieces
Phone straps:
??? pieces
Individual stall
Your total income
Income from the individual stall +
Income from the social enterprise
= $100 + $260 = $360
Income from the social enterprise :
($15 x 208) / 12 = $260

Example 4

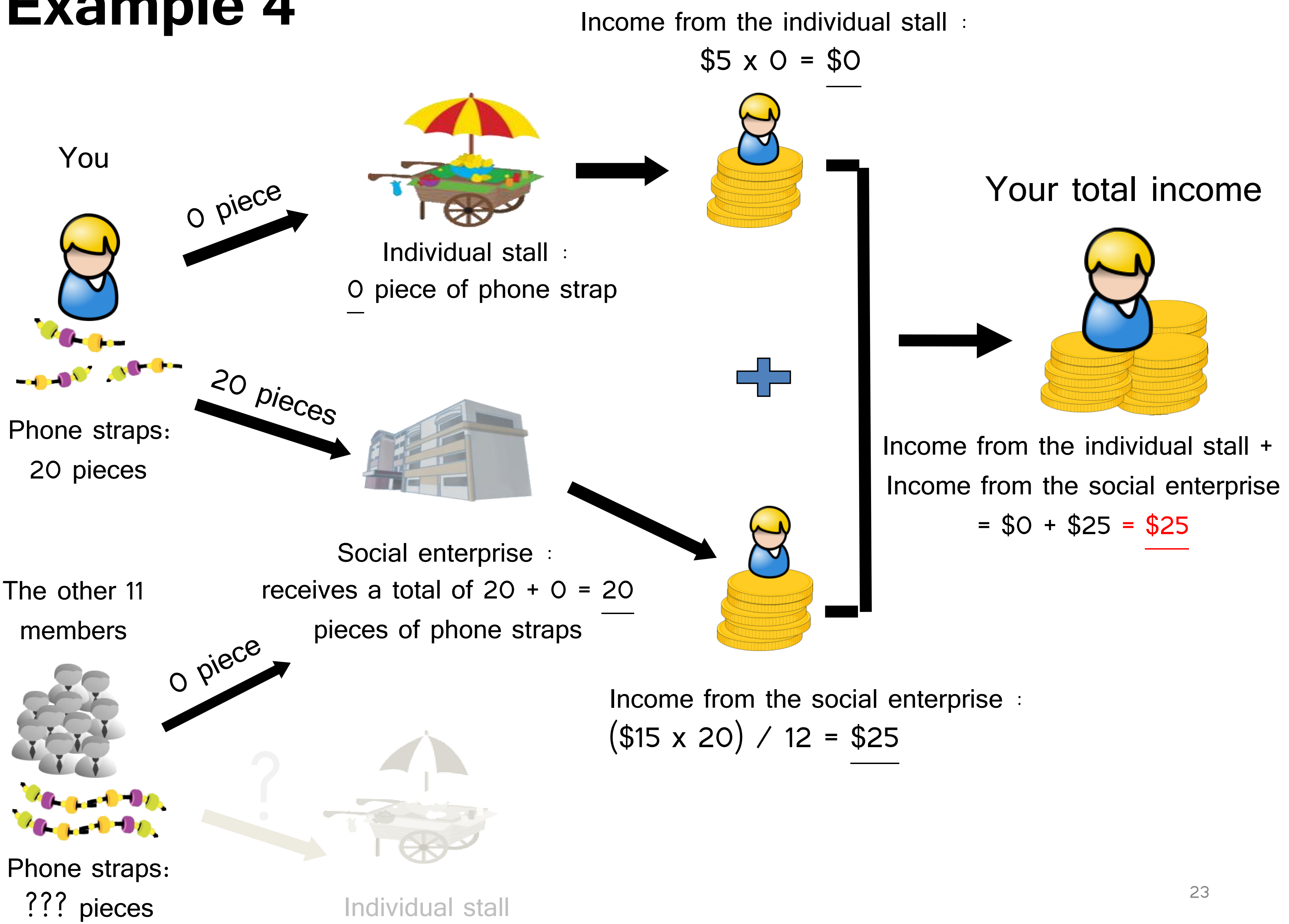
Income from the individual stall :
$5 x 0 = $0
You
0 piece
Individual stall :
0 piece of phone strap
Your total income
Phone straps:
20 pieces
20 pieces
Income from the individual stall +
Income from the social enterprise
= $0 + $25 = $25
Social enterprise :
receives a total of 20 + 0 = 20
pieces of phone straps
The other 11
members
0 piece
Income from the social enterprise :
($15 x 20) / 12 = $25
Phone straps:
??? pieces
Individual stall

Rules of manufacturing the phone straps

- Half of the members: Large beads and 6 minutes
  The other half of the members: Small beads and 3 minutes

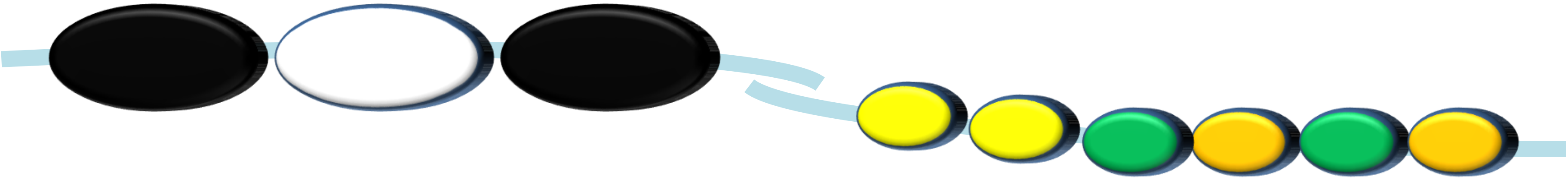

- The members with less time will need to complete some translation exercises in the remaining 3 minutes
- Put the finished product into the transparent box

- Goals: (1) **Manufacture the highest number of phone straps**
  (2) Allocate the phone straps to the individual stall and the social enterprise **to obtain the highest profit**
