# Supplementary material for: The effect of subgroup homogeneity of efficacy on contribution in public good dilemmas
Source: PLoS One. 2018 Jul 31;13(7):e0201473. doi: 10.1371/journal.pone.0201473 (PMC6067760; doi:10.1371/journal.pone.0201473)
Supplement: S2 Appendix — (DOCX) [file pone.0201473.s002.docx]

**S2 Appendix. Computation of bonus payments.**

After the experiment ended, the experimenter randomly selected one out of six participants to receive a bonus which was equal to his/her actual earning in the task. The participant receiving the bonus was told the total contributions of all 12 participants (say X cell phone straps), which included those of six participants participating in the current session and those in a previous session. Say the participant kept Y straps in his/her private account. The participant would receive X * $15 / 12 + Y * $5 as a bonus in addition to the $50 participation fee. The other five participants received only the flat rate of HK$50 for participation.

A distribution of the earnings of participants in the task is shown below.


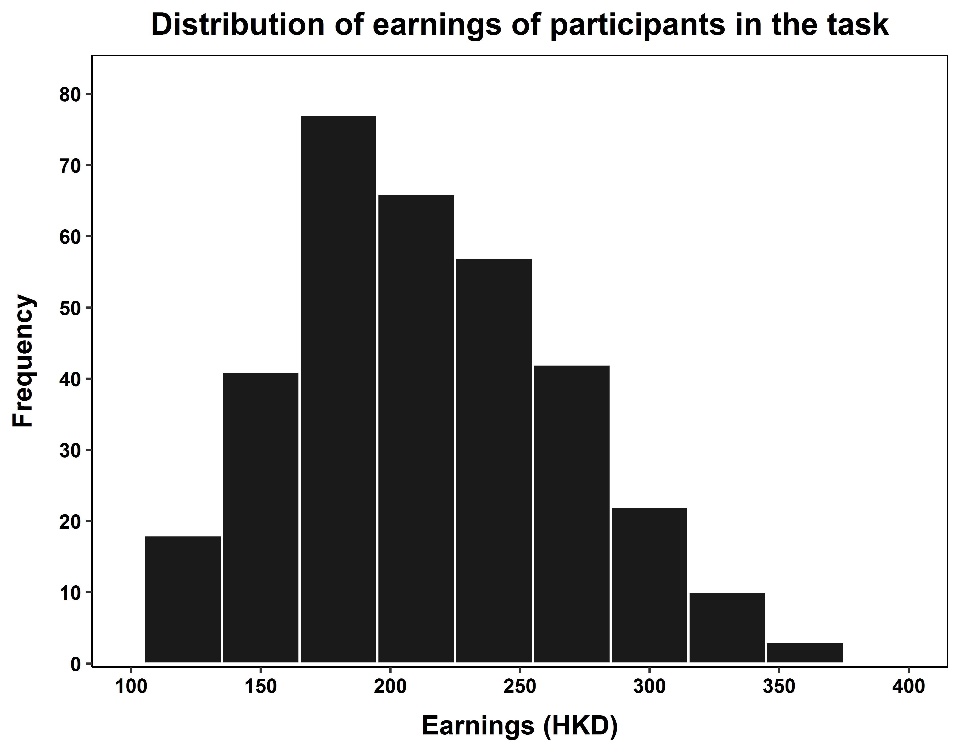


For participants joining the first session, their contributions were pooled with those participants in pilot experiment sessions.

In pilot sessions, participants did not receive any payments and they were told that their contributions would be pooled with participants in *other* sessions. Because they were not paid, they did not (need to) receive feedback regarding the contributions of participants in other sessions.
