# Supplementary material for: The effect of subgroup homogeneity of efficacy on contribution in public good dilemmas
Source: PLoS One. 2018 Jul 31;13(7):e0201473. doi: 10.1371/journal.pone.0201473 (PMC6067760; doi:10.1371/journal.pone.0201473)
Supplement: S3 Appendix — (DOCX) [file pone.0201473.s003.docx]

**S3 Appendix. Mplus code of the multilevel moderated mediation model.**

**TITLE:**

Subgroup Homogeneity Moderated Mediation Analysis

**DATA:**

File = Beads Data Full Mplus.csv;

Listwise = ON;

**VARIABLE:**

MISSING are all (999);

NAMES = ID SGroupID Gender GrpHomo Efficacy numHighE numLowE PEff_sg PEff_se exSGpCon exSECon StrMade StrSelf StrCon ConRate;

*! Variable explanation: ID = Participant ID, SGroupNo = Subgroup ID, Gender = Participant's gender, GrpHomo = Subgroup homogeneity (0 = Homogeneous; 1 = Heterogeneous), Efficacy = Manipulation of efficacy (0 = Low-efficacy; 1 = High-efficacy), numHighE = Number of high efficacy individuals in the subgroup, numLowE = Number of low efficacy individuals in the subgroup, PEff_sg = Perceived efficacy relative to subgroup, PEff_se = Perceived efficacy relative to social enterprise, exSGpCon = Expectation of subgroup contribution, exSECon = Expectation of social enterprise contribution, StrMade = Strap production, StrSelf = Straps not contributed, StrCon = Straps contributed, ConRate = proportion of contribution*

USEVARIABLES = SGroupID GrpHomo Efficacy PEff_sg exSGpCon StrCon;

*! Variables used in this analysis*

WITHIN = Efficacy; *! Level-1 variable*

BETWEEN = GrpHomo; *! Level-2 variable*

CLUSTER = SGroupID; *! Cluster variable*

**ANALYSIS:**

TYPE = TWOLEVEL RANDOM;

*! Multilevel model with random intercepts and random slopes*

ESTIMATOR=ML;

*! Maximum likelihood estimation*

**MODEL:**

%WITHIN% *! Level-1 part of the model*

s_a1 | PEff_sg ON Efficacy;

s_a2 | exSGpCon ON Efficacy;

s_b1 | StrCon ON PEff_sg;

s_b2 | StrCon ON exSGpCon;

s_c | StrCon ON Efficacy;

*! Specifies random slopes of the mediation model*

%BETWEEN% *! Level-2 part of the model*

[s_a1] (a1);

[s_a2] (a2);

[s_b1] (b1);

[s_b2] (b2);

[s_c] (c);

*! Naming the random slopes for model constraint part*

StrCon on GrpHomo;

PEff_sg on GrpHomo (p1);

exSGpCon on GrpHomo (p2);

s_a1 on GrpHomo (m1);

s_a2 on GrpHomo (m2);

s_c on GrpHomo;

*! Specifies paths for subgroup homogeneity as moderator*

PEff_sg exSGpCon WITH StrCon;

PEff_sg exSGpCon WITH s_a1 s_a2 s_b1 s_b2 s_c;

StrCon WITH s_a1 s_a2 s_b1 s_b2 s_c;

s_a1 WITH s_b1 (covab1);

s_a2 WITH s_b2 (covab2);

s_a1 WITH s_a2 s_b2 s_c;

s_a2 WITH s_b1 s_c;

s_b1 WITH s_b2 s_c;

s_b2 WITH s_c;

*! Estimating covariances of the variables*

**MODEL CONSTRAINT:**

NEW(LowEff HighEff Homo Hetero sim1a sim1b sim2a sim2b ind1a ind1b ind2a ind2b ind3a ind3b ind4a ind4b mome1 mome2);

*! Define new parameters*

LowEff = 0;

HighEff = 1;

Homo = 0;

Hetero = 1;

sim1a = p1+m1*LowEff;

*! Effect of subgroup homogeneity on perceived efficacy for low-efficacy individuals*

sim1b = p1+m1*HighEff;

*! Effect of subgroup homogeneity on perceived efficacy for high-efficacy individuals*

sim2a = p2+m2*LowEff;

*! Effect of subgroup homogeneity on expectation of contribution for low-efficacy* individuals

sim2b = p2+m2*HighEff;

*! Effect of subgroup homogeneity on expectation of contribution for high-efficacy individuals*

ind1a = (p1+m1*LowEff)*b1;

*! Conditional indirect effect of subgroup homogeneity on contribution through perceived efficacy for low-efficacy individuals*

ind1b = (p1+m1*HighEff)*b1;

*! Conditional indirect effect of subgroup homogeneity on contribution through perceived efficacy for high-efficacy individuals*

ind2a = (p2+m2*LowEff)*b2;

*! Conditional indirect effect of subgroup homogeneity on contribution through expectation of contribution for low-efficacy individuals*

ind2b = (p2+m2*HighEff)*b2;

*! Conditional indirect effect of subgroup homogeneity on contribution through expectation of contribution for high-efficacy individuals*

ind3a = (a1+m1*Homo)* b1 + covab1;

*! Conditional indirect effect of efficacy on contribution through perceived efficacy for homogeneous subgroups*

ind3b = (a1+m1*Hetero)* b1 + covab1;

*! Conditional indirect effect of efficacy on contribution through perceived efficacy for heterogeneous subgroups*

ind4a = (a2+m2*Homo)* b2 + covab2;

*! Conditional indirect effect of efficacy on contribution through expectation of contribution for homogeneous subgroups*

ind4b = (a2+m2*Hetero)* b2 + covab2;

*! Conditional indirect effect of efficacy on contribution through expectation of contribution for heterogeneous subgroups*

mome1 = m1*b1;

mome2 = m2*b2;

*! Index of moderated mediation*

**OUTPUT:**

SAMPSTAT; *! Generate sample statistics*

CINTERVAL; *! Generate confidence intervals*
