# Supplementary material for: The effect of subgroup homogeneity of efficacy on contribution in public good dilemmas
Source: PLoS One. 2018 Jul 31;13(7):e0201473. doi: 10.1371/journal.pone.0201473 (PMC6067760; doi:10.1371/journal.pone.0201473)
Supplement: S4 Appendix — (DOCX) [file pone.0201473.s004.docx]

**S4 Appendix. Additional data analyses on subgroup configurations.**

We conducted additional data analyses to test the differences in strap production and contribution among the four subgroup configurations: Three high-efficacy members (3H), two high-efficacy members and one low-efficacy member (2H1L), one high-efficacy member and two low-efficacy members (1H2L), and three low-efficacy members (3L).

We conducted ANOVAs with subgroup configuration as independent variable, and strap production and contribution of subgroups as dependent variables. There were 112 observations in these analyses because there was a total of 112 subgroups.

Strap production:

There was a significant difference in strap production among different subgroup configurations, *F* (3, 108) = 825.25, *p* < .001. To test the pairwise differences in strap production, post hoc tests with Tukey HSD correction were performed. 3H subgroups produced more straps than 2H1L subgroups, *M* = 103.55 vs. 83.15, *p* < .001. 2H1L subgroups produced more straps than 1H2L subgroups, *M* = 83.15 vs. 58.26, *p* < .001. 1H2L subgroups produced more straps than 3L subgroups, *M* = 58.26 vs. 35.03, *p* < .001.

Strap contribution:

There was a significant difference in strap contribution among different subgroup configurations, *F* (3, 108) = 37.08, *p* < .001. To test the pairwise differences in strap contribution, post hoc tests with Tukey HSD correction were performed. 3H subgroups contributed more straps than 2H1L subgroups, *M* = 48.21 vs. 32.70, *p* < .001. However, 2H1L subgroups did not contribute significantly more straps than 1H2L subgroups, *M* = 32.70 vs. 25.07, *p* < .13. 1H2L subgroups contributed more straps than 3L subgroups, *M* = 25.07 vs. 13.79, *p* < .01.

| **Means (Standard deviations) of strap production and strap contribution across different subgroup configurations (*N*=112).** | | | | |
| --- | --- | --- | --- | --- |
|  | Subgroup Configurations | | | |
|  | 3H | 2H1L | 1H2L | 3L |
|  |  |  |  |  |
|  | (*N* = 29) | (*N* = 27) | (*N* = 27) | (*N* = 29) |
| Production | 103.55 (6.38) | 83.15 (6.79) | 58.26 (5.19) | 35.03 (3.27) |
| Contribution | 48.21 (15.05) | 32.70 (15.70) | 25.07 (12.11) | 13.79 (5.95) |
|  |  |  |  |  |
